# Refining α-synuclein seed amplification assays to distinguish Parkinson’s disease from multiple system atrophy

**DOI:** 10.1186/s40035-025-00469-6

**Published:** 2025-02-07

**Authors:** James A. Wiseman, Clinton P. Turner, Richard L. M. Faull, Glenda M. Halliday, Birger Victor Dieriks

**Affiliations:** 1 Department of Anatomy and Medical Imaging, University of Auckland, 85 Park Road, Grafton, Auckland, 1142 New Zealand; 2 Centre for Brain Research, University of Auckland, Auckland, 1023 New Zealand; 3 Brain and Mind Centre & Faculty of Medicine and Health School of Medical Sciences, The University of Sydney, Sydney, NSW 2050 Australia; 4 LabPlus, Department of Anatomical Pathology, Te Whatu Ora, Auckland, New Zealand; 5 Neuroscience Research Australia & Faculty of Medicine School of Medical Sciences, University of New South Wales, Sydney, NSW 2052 Australia

**Keywords:** α-Synuclein, Parkinson’s disease, Multiple system atrophy, Seed amplification assays, α-Synuclein strains, Conformational variability, RT-QuIC

## Abstract

**Background:**

Parkinson’s disease (PD) and multiple system atrophy (MSA) are two distinct α-synucleinopathies traditionally differentiated through clinical symptoms. Early diagnosis of MSA is problematic, and seed amplification assays (SAAs), such as real-time quaking-induced conversion (RT-QuIC), offer the potential to distinguish these diseases through their underlying α-synuclein (α-Syn) pathology and proteoforms. Currently, SAAs provide a binary result, signifying either the presence or absence of α-Syn seeds. To enhance the diagnostic potential and biological relevance of these assays, there is a pressing need to incorporate quantification and stratification of α-Syn proteoform-specific aggregation kinetics into current SAA pipelines.

**Methods:**

Optimal RT-QuIC assay conditions for α-Syn seeds extracted from PD and MSA patient brains were determined, and assay kinetics were assessed for α-Syn seeds from different pathologically relevant brain regions (medulla, substantia nigra, hippocampus, middle temporal gyrus, and cerebellum). The conformational profiles of disease- and region-specific α-Syn proteoforms were determined by subjecting the amplified reaction products to concentration-dependent proteolytic digestion with proteinase K.

**Results:**

Using our protocol, PD and MSA could be accurately delineated using proteoform-specific aggregation kinetics, including α-Syn aggregation rate, maximum relative fluorescence, the gradient of amplification, and core protofilament size. MSA cases yielded significantly higher values than PD cases across all four kinetic parameters in brain tissues, with the MSA-cerebellar phenotype having higher maximum relative fluorescence than the MSA-Parkinsonian phenotype. Statistical significance was maintained when the data were analysed regionally and when all regions were grouped.

**Conclusions:**

Our RT-QuIC protocol and analysis pipeline can distinguish between PD and MSA, and between MSA phenotypes. MSA α-Syn seeds induce faster propagation and exhibit higher aggregation kinetics than PD α-Syn, mirroring the biological differences observed in brain tissue. With further validation of these quantitative parameters, we propose that SAAs could advance from a yes/no diagnostic to a theranostic biomarker that could be utilised in developing therapeutics.

**Supplementary Information:**

The online version contains supplementary material available at 10.1186/s40035-025-00469-6.

## Introduction

Parkinson’s disease (PD) and multiple system atrophy (MSA) are neurodegenerative disorders that share a common pathological hallmark: the aberrant accumulation of misfolded α-synuclein (α-Syn) protein within the central nervous system [1–9]. Despite their shared α-Syn pathology, these diseases exhibit substantial pathological heterogeneity in specific brain areas–both within and between diseases–as well as differential clinical presentations, rates of progression, and responses to treatments.

Both diseases have α-Syn aggregates with differences in their proteoform structure, with MSA having multiple types of structures identified [10–14]. PD and MSA are currently diagnosed based on their differentiating clinical features [15, 16], but this is about to change with a push to identify these patients in life according to their underlying α-Syn pathology [17, 18]. The potential of seed amplification assays (SAAs) to reliably diagnose α-synucleinopathies early in their pathogenesis has garnered significant research interest. This has particular significance for those with early or prodromal disease where clinical symptoms are ambiguous. Early identification of these conditions is crucial, as significant irreversible neurodegeneration typically occurs during the long prodromal periods associated with these diseases; this limits the therapeutic impact of current treatments at the stage clinical symptoms manifest, and a clinical diagnosis can be made [19–22]. With the rising ante-mortem diagnostic potential of SAAs, detecting and understanding the differences in brain α-Syn proteoforms are now paramount.

SAAs, such as real-time quaking-induced conversion (RT-QuIC), can accurately detect α-Syn seeding activity with high specificity and sensitivity (> 90%) in a diversity of biological samples from the human brain with PD and MSA [23–29]. Presently, SAAs yield a binary outcome, indicating the presence or absence of α-Syn seeds. Further development to incorporate quantification and stratification of α-Syn proteoform-specific aggregation kinetics into current SAA pipelines is necessary to provide more precise disease-specific diagnostic insights into the progression and severity of tissue pathologies.

There is now some evidence that SAA quantitative data output can provide a reliable correlation for the underlying biological activity of α-Syn proteoforms in the human brain beyond that of a yes/no diagnostic readout. Specifically, the length of the lag phase (defined as the time taken to reach the amplification threshold) has been suggested as the most promising variable correlating with the original pathological α-Syn seed concentration [14, 30, 31]. Significant challenges remain, however, due to the considerable heterogeneity among SAA-based studies, which is primarily driven by the absence of a standardised SAA protocol within the field. Factors influencing SAA aggregation kinetics include the nature of the monomeric α-Syn substrate, buffer pH levels, initial seed concentration, ambient temperature, the presence of biological contaminants, shaking dynamics, and the ionic makeup of reaction buffers (reviewed in [32, 33]). The considerable impact of these micro-environmental factors on assay outcomes underscores the critical need for the standardisation of SAA assay parameters and the customisation of buffer compositions to permit biologically relevant SAA readouts.

Studies using SAAs to identify different proteoforms between PD and MSA have been scarce. For instance, Shahnawaz et al*.* (2020) found distinct α-Syn profiles for PD and MSA in cerebrospinal fluid (CSF) samples, highlighting differences in fluorescence intensity and resistance to proteolytic digestion. In contrast, studies conducted by van der Perren et al*.* (2020) and Luan et al*.* (2022) demonstrated similar fluorescence intensities for PD and MSA patient-derived α-Syn from human brain tissue and saliva, respectively. Rossi et al. (2020) reported that RT-QuIC effectively detected pathological α-Syn in CSF samples from PD and dementia with Lewy bodies but not from MSA patients. To add a further layer of complexity, the seeding activity of aggregated α-Syn has been shown to vary across different regions of the human brain with PD [34] and MSA [23]. This variability highlights the challenge of consistently differentiating α-synucleinopathies with SAAs and underscores the need to finetune/standardise assay methodologies.

Although these studies are encouraging, elucidating the biological relevance of SAA-based diagnostics has proven far more challenging. The present study used RT-QuIC to provide a comprehensive analysis of the aggregation kinetics of α-Syn derived from different pathologically relevant brain regions of neuropathologically confirmed cases of PD and MSA. Amplified reaction products were subjected to concentration-dependent proteolytic digestion with proteinase K (PK) to obtain insights into the conformational profile of disease- and region-specific α-Syn proteoforms.

## Materials and methods

### Human brain tissue and CSF samples

Human post-mortem brain tissues used in this study were received from (1) the Sydney Brain Bank (MSA and neurologically normal cases) and (2) the Neurological Foundation Human Brain Bank (New Zealand; PD and neurologically normal cases). All brain tissues were donated with written informed consent from donors and their families, and all protocols were approved by the University of Sydney Human Research Ethics Committee (2019/491) and the University of Auckland Human Participants Ethics Committee (Ref: 011654). CSF was collected post-mortem. To minimise any confounding influence that blood might have on the aggregation kinetics of CSF samples, all samples were centrifuged for 10 min at 720 × *g*. The supernatant was collected and aliquoted for subsequent seeding of RT-QuIC reactions. All experiments were conducted following relevant guidelines and regulations. A neuropathologist assessed all cases used in this study; detailed information of all cases used in this study is presented in Table S1. All PD cases (*n* = 10) had a clinical history of PD, and pathological features were consistent with PD pathology, as confirmed by a neuropathologist (Table 1). Key neuropathological features were loss of pigment and pigmented cells in the substantia nigra and accumulation of Lewy pathology in the substantia nigra and other brain regions; many cases also had evidence of cortical Lewy body disease. All MSA cases (*n* = 10) had a clinical history of MSA-parkinsonian phenotype (MSA-P; *n* = 5) or MSA-cerebellar phenotype (MSA-C; *n* = 5), and pathological features were consistent with MSA pathology as confirmed by a neuropathologist (Table 1). Key neuropathological features were the presence of α-Syn-immunopositive neuronal and glial cytoplasmic inclusions in the brainstem, basal ganglia, cerebellum, and hippocampus. The neurologically normal cases (*n* = 5) had no clinical history of neurological abnormalities, and no other significant neuropathology was noted upon post-mortem examination (Table 1, Table S1).

**Table 1:** Summary of case statistics for human brain tissues used in this study

	**MSA**	**PD**	**Neurologically Normal**
*N*	10 (MSA-P, *n* = 5; MSA-C, *n* = 5)	10	5
Disease duration (years) Mean ± SD	10.1 ± 5.1	17.1 ± 7.1	–
Age (years) Mean ± SD	67.7 ± 8.5	74.7 ± 8.9	81.8 ± 11.6
Post-mortem delay (h) Mean ± SD	24.6 ± 11.6	10.0 ± 6.2	26.2 ± 7

### Seed amplification assay

The described seed amplification protocol is an in-house protocol based on a previously described protocol [35]. The SAA used in this study was optimised for use with fresh-frozen post-mortem human brain tissues and utilised RT-QuIC to amplify and detect seeded α-Syn aggregate conformations.

### Buffer preparation

All buffers presented in Table 2 were prepared and filter-sterilised using a 0.22 µm filter prior to commencing the SAA. All buffers were discarded after three months. Briefly, the homogenisation buffer for the homogenisation of fresh-frozen human brain tissue contained 0.1% (*w*/*v*) SDS prepared in PBS pH 7.4. The homogenisation buffer was prepared in a 50 mL conical tube, mixed for 30 s using a Vortex-Genie 2 (Scientific Industries) at level 10, and stored at 4 °C. The base mix was prepared in a 50 mL conical tube. To avoid errors in concentration caused by the pipetting of small volumes, the base mix was typically prepared for 500 reactions at a time (Table 2 details the preparation for five reactions). For five reactions, 217 µL of ddH_2_O, 100 µL of 5 × PBS pH 6.9, 17 µL of 5 M NaCl, and 1 µL of 0.5 M EDTA were added together and vortex-mixed.

**Table 2:** Reagent set-up for real-time quaking-induced conversion

Component	Volume	Final concentration
**Homogenisation buffer.** Filter-sterilised and stored in a 50 mL conical tube. Can be stored at 4 °C for up to 3 months.		
SDS	0.05 g	0.1% (*w*/*v*)
1 × PBS pH 7.4	50 mL	
**Thioflavin-T solution.** Prepared in conical tube and filter-sterilised. 10 mmol/L stock solution diluted to 1 mmol/L working solution. Can be stored in the dark at 4 °C or -20 °C for up to 3 months. Working solution should be discarded after use.		
Thioflavin-T	0.036 g	10 mmol/L
ddH_2_O	10 mL	–
**5** × **Phosphate buffered saline.** Made up in 1L stock batch. Filter-sterilised and stored in a 50 mL conical tube. Can be stored at 4 °C for up to 3 months.		
NaCl KCl Na_2_HPO_4_ KH_2_PO_4_	80 g 2 g 14.2 2.4 g	1.37 mol/L 26.8 mmol/L 0.1 mol/L 17.6 mmol/L
**Base mix.** *For 5 reactions.* Prepared in a conical tube and filter-sterilised. Can be stored at -20 °C for up to 3 months.		
ddH_2_O	217 µL	–
5 × PBS pH 6.9	100 µL	1.49 ×
5 M NaCl	17 µL	254 mmol/L
0.5 M EDTA	1 µL	1.49 mmol/L
**Final reaction mix.** Prepared in a conical tube. Prepare fresh for each experiment and gently mix using a P1000 pipette.		
Base Mix	57 µL	1 × PBS, 170 mmol/L NaCl, 1 mmol/L EDTA
Thioflavin-T (1 mmol/L)	1 µL	10 µmol/L
Recombinant α-Syn monomer	27 µL	0.10 mg/mL
		Note: total concentration of NaCl in final mix (PBS + 5 mol/L NaCl = 444.4 mmol/L)

### Fresh-frozen tissue homogenisation

Human brain tissue samples were transferred into microcentrifuge tubes using autoclaved stainless-steel tweezers. All samples were weighed at room temperature using an electronic balance (Mettler Toledo, AB54) and diluted to a final concentration of 0.625% (*w*/*v*) in homogenisation buffer. Samples were placed in a Bullet Blender® Homogeniser (Next Advance; Troy, NY) and subjected to two rounds of tissue homogenisation for 4 min each on level 8. All samples were inspected for potential leakage between the two homogenisation rounds, and screw caps were tightened or replaced as necessary. Samples were centrifuged at 10,000 × *g* for 10 min following homogenisation. The supernatant from each sample, which contained the soluble fraction, was then carefully pipetted and aliquoted into 600 µL Eppendorf tubes and stored at − 80 °C for subsequent use. The remaining insoluble fraction from each sample, contained within the pellet, was also retained and stored at − 80 °C for use in downstream investigations.

### CSF collection and preparation

CSF samples were collected directly from the choroid plexus and lateral ventricles during processing of post-mortem human brains. CSF samples were aliquoted and diluted 1:2 in homogenisation buffer and stored at − 80 °C for downstream analysis.

### RT-QuIC

All tissue samples were thawed at room temperature and vortex-mixed for 30 s. The 10 mg/mL stocks of human α-Syn monomers (RP-003, Proteos, Kalamazoo, MI) were thawed on ice, diluted to 5 mg/mL in filtered PBS (pH 7.4) and stored in 25 µL aliquots at − 80 °C. Aliquots of monomeric α-Syn were thawed on ice as required to minimise the risk of spontaneous aggregation due to thawing at higher temperatures. Monomeric α-Syn was diluted to a working concentration of 0.37 mg/mL in filtered PBS (pH 7.4), loaded into 100-kDa filters (88503, ThermoFisher Scientific, Waltham, MA) and centrifuged at room temperature (5 min at 1350 × *g*; 5424-R, Eppendorf, Hamburg, Germany) to remove any existing aggregates. Filters were discarded and the volume of the filtrate was carefully measured using a P1000 pipette. Approximately 10% of the monomeric solution is lost during filtration; the resulting filtrate volume usually accommodates 11 reactions (per aliquot of monomeric α-Syn). 85 µL of the final reaction buffer (Table 2) was then pipetted into each reaction well of a 96-well Optical-Bottom plate (165305, Nunc, Roskilde, Sjelland, Denmark), and 15 µL (final concentration ~ 1 × 10^–3^) of the desired tissue (or CSF) sample was gently mixed using a P200 pipette. Care was taken to ensure no air bubbles were introduced during plate loading, as these can lead to variable results. The final reaction buffer was always freshly prepared and mixed gently by triturating three times with a P1000 pipette. More aggressive mixing techniques can induce spontaneous aggregation of monomeric α-Syn. All samples were loaded in triplicate, and samples that yielded fluorescent signals above 10 [4] relative fluorescent units (r.f.u) in at least two of the three sample repeats were considered positive. All disease-specific samples from the same region were run in the same assay; sample preparation and all reaction conditions were kept identical between individual assay runs to minimise potential variability. All assays included stringent region-matched controls sampled from neurologically normal patient brains. Control samples were also run in triplicate.

It is important to highlight that in MSA, seeding activity is independent of α-Syn solubility [23, 36], while activity of PD α-Syn consists predominantly of detergent-insoluble species, which potentially reduces the available pool of α-Syn seeds [36]. These data, however, are primarily based on the solubility of α-Syn in weaker detergents, namely sarkosyl and Triton-X, whereas the present study utilised SDS, which more effectively denatures and solubilises α-Syn [37]. Due to this enhanced solubility, we elected to utilise the soluble fraction in all experiments, which we would expect to contain a greater proportion of α-Syn species than would be present if sarkosyl or Triton-X had been used.

Once loaded, the plate was covered with adhesive sealing tape (MicroAmp, 4311971) and inserted into the plate carrier of a CLARIOstar plate reader (BMG Labtech, Ortenberg, Germany). Briefly, the assay was conducted at 42 °C for a total duration of 60 h, which consisted of alternating 1-min cycles of double-orbital shaking followed by stationary incubation. Fluorescent readings were measured every 10 min. This increased the kinetic resolution of the data output compared to 30-min cycles. This was particularly important for MSA cases, which were characterised by significantly higher protein aggregation rates (PAR) and gradients of amplification. The optimal cycle time of 10 min provided a sufficient temporal resolution to accurately resolve subtle differences between all tissue samples while ensuring manageable data loads. Optimisation and final assays were conducted across two different laboratories (The University of Auckland and The University of Sydney) using identical machines, settings, and buffers.

Optical settings were manually configured to ensure a spectral range between 445–﻿10 (excitation wavelength = 445 ± 5 nm) and 480–10 (emission wavelength = 480 ± 5 nm); a dichroic value of 462.5 was selected. After the assay, RT-QuIC reaction products from each well were transferred into individual Eppendorf tubes and stored at − 20 °C for subsequent conformational characterisation. All amplification products were handled in a fume hood with appropriate personal protective equipment. All RT-QuIC assays included stringent positive and negative controls. Positive controls consisted of PD/MSA patient-derived brain tissue or recombinant α-Syn aggregates. Negative controls consisted of PBS (pH 7.4) and homogenisation buffer. Region-matched neurologically normal control human brain tissue samples were also included in all RT-QuIC assays (Fig. S1), in addition to secondary control middle temporal gyrus samples.

### Proteolytic digestion of amplified α-Syn aggregates

The conformational profiles of RT-QuIC-amplified samples were interrogated using concentration-dependent proteolytic digestion with PK (3115828001, Roche, Basel, Switzerland). Briefly, 10 µL of samples containing RT-QuIC-amplified α-Syn aggregates were treated with different PK concentrations (final concentration 0, 1, 10, 100 μg/mL) and incubated at 37 °C for 60 min. Proteolytic digestion was arrested by heating the samples in NuPage LDS sample buffer (ThermoFisher Scientific, NP0007) at 95 °C for 10 min. All incubations were conducted in a fume hood. Then 20 µL of each digested product was loaded into 4%–12% Bis–Tris gels (ThermoFisher Scientific, NP0336BOX) to be resolved via sodium dodecyl sulphate–polyacrylamide gel electrophoresis , following the manufacturer’s protocol. Gels were run using an MES SDS running buffer (ThermoFisher Scientific, NP0002) with a Spectra™ Multicolor Low Range Protein Ladder (ThermoFisher Scientific, 26628) to resolve low-molecular-weight proteins. The running buffer was placed in a -20 °C freezer 1 h before use to prevent overheating in gel runs. Gels were run for 30 min, applying a constant voltage of 175 V. Once protein migration was complete, gels were washed (3 × 10 min in ddH_2_O) and stained with Coomassie (SimplyBlue™ Safe Stain; ThermoFisher Scientific, LC6060) for 2 h with gentle rocking. Gels were then washed overnight in 150 mL of ddH_2_O with 30 mL of 20% (*v*/*v*) NaCl added to enhance the sensitivity of the Coomassie stain. Gels were washed a final time in 150 mL of ddH_2_O for 5 h, and images were acquired using a Gel Doc™ EZ Imager system (BioRad, Hercules, CA). All quantifications of α-Syn digestion were performed in ImageLab (BioRad). For each lane, the mean molecular weight across all digestion bands was calculated and normalised to the 0 μg/mL PK condition so that all samples were internally normalised.

### Data analysis

**Fig. 1:**
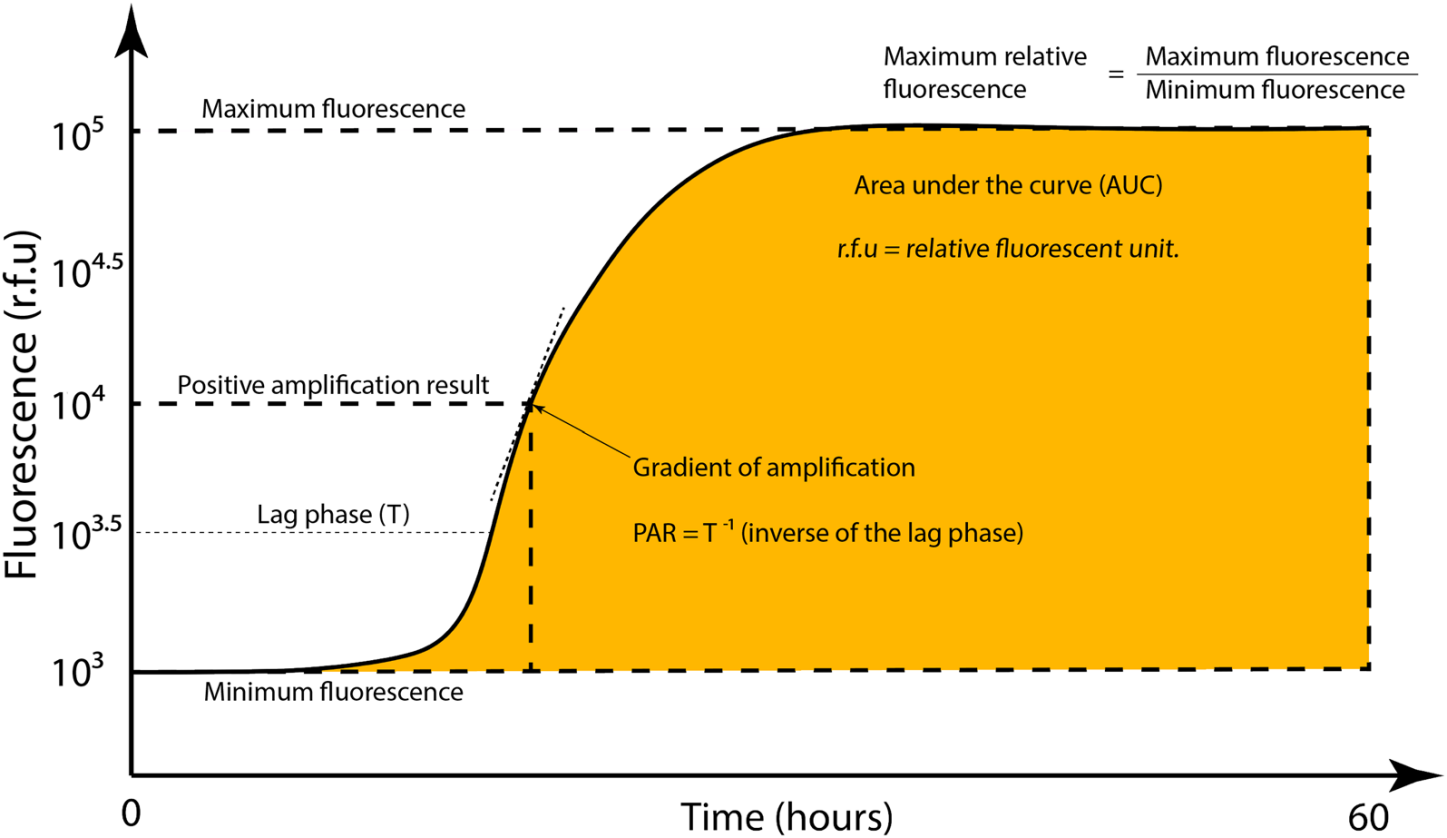
Schematic diagram defining the key analytical measures quantified in this study.

The amplification curves for all amplified samples were plotted and analysed. The key analytical measures determined were (Fig. 1):

- Length of lag phase (T, defined as the time taken to reach the amplification threshold). The amplification threshold was set to 10^3.5^ r.f.u to ensure consistency across samples.
- Protein aggregation rate (PAR, defined as the inverse of the lag phase, T^−1^).
- Area under the curve (AUC, r.f.u × T). The area under the curve was approximated by fitting a sigmoidal function to each amplification curve. The integral of each sigmoidal function was then determined using the trapezoidal method.
- Gradient of amplification. The gradient of amplification was determined by calculating the gradient of the tangent at the inflection point of each amplification curve.
- Maximum fluorescence. The maximum fluorescence was determined by calculating the average of the highest 10 r.f.u values for each case.
- Minimum fluorescence. The minimum fluorescence was determined by calculating the average of the lowest 10 r.f.u values for each case. The first fluorescent read-out (pre-quaking) was always omitted, as it was often an outlier and was technically measured prior to assay commencement.
- Maximum relative fluorescence. The maximum relative fluorescence was determined by calculating a ratio of the maximum to the minimum fluorescence; this relative maximum fluorescence value was utilised in all subsequent comparisons. All maximum fluorescence quantifications were internally normalised to the pre-amplification minimum fluorescence to ensure accurate comparisons between different conditions.

Data visualisation and statistical analysis were performed using RStudio (R version 4.3.1). All data are presented as mean ± standard deviation (SD). The Shapiro–Wilk test was used to assess data distribution. Parametric tests were utilised when data were normally distributed, and non-parametric tests were performed when data did not assume a normal distribution. A one-way analysis of variance (ANOVA), with Tukey’s multiple comparison adjustment, was used when comparing multiple groups. An unpaired *t*-test or Mann–Whitney U test was used when comparing two groups. Statistical significance was set as *P* < 0.05. In all quantification figures: ***P < 0.001, **P < 0.01, *P < 0.05.

## Results

### Optimisation of RT-QuIC and PK digestion assays

Several key aspects of the described RT-QuIC assay and PK digestion protocol required careful optimisation to ensure the highest reproducibility and accuracy of the results detailed in this study. The pertinent areas requiring optimisation within the overall workflow are detailed in Supplementary Fig. S2. As previously shown [38], α-Syn pathology load varies substantially between PD and MSA, and between respective regions. As the current study involved pathologically burdened regions from both PD and MSA patient cases, it was essential to determine an initial seed concentration to maximise the seeding efficiency across all samples involved. To achieve this, each PD and MSA region was systematically titrated to determine the upper and lower concentration limits at which effective seeding was compromised. A tissue homogenate concentration ranging from 0.5% to 10% (*w*/*v*) was utilised. In general, tissue with high pathology load failed to propagate seeds at higher seed concentrations, presumably due to the impact of molecular overcrowding, which prevented available α-Syn seeds from efficiently inducing further misfolding of monomeric α-Syn. Tissue with low pathology loads typically had better seeding efficiency at higher seed concentrations but often failed to seed aggregation at lower seed concentrations.

The optimisation of tissue homogenate concentration was also important due to its effects on aggregation rate. The aggregation rate was defined as the inverse of the lag phase, which provides a robust measurement of the time taken to reach the minimal aggregation threshold, after which the growth phase is initiated. If the aggregation rate is too high (short lag phase) across interrogated samples, resolving the more subtle kinetic differences between samples is difficult. A concentration of 0.625% (*w*/*v*) was chosen to guarantee efficient aggregation in all tissue samples studied in the present study. This concentration also ensured optimal temporal resolution of the aggregation rate, enabling clear differentiation of the aggregation kinetics both within and between PD and MSA brain regions. Rigorous secondary validations were performed to confirm this optimal concentration point prior to collecting the data presented in this study (Fig. S3).

### Differential aggregation kinetics between PD and MSA patient-derived α-Syn

**Fig. 2:**
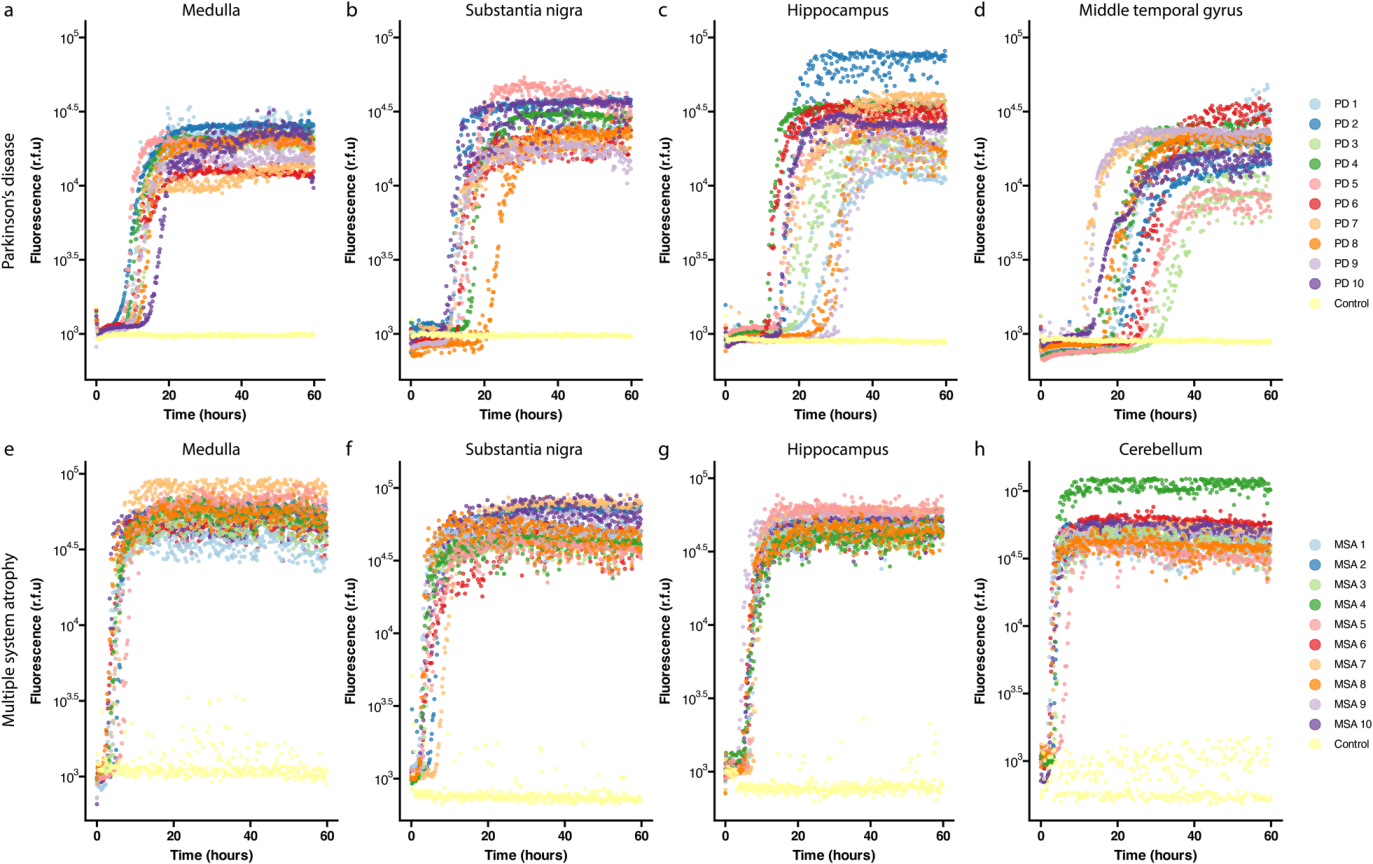
RT-QuIC amplification curves of PD and MSA patient-derived brain tissues. Data are the triplicate mean for each case. Representative individual repeats are displayed in Figure S7.

To investigate differential seeding propensities between PD and MSA patient-derived α-Syn, RT-QuIC was conducted on patient-derived tissue homogenates sampled from the medulla, substantia nigra, and hippocampus of PD and MSA patients. The middle temporal gyrus from PD patients and the cerebellum from MSA patients were also investigated due to their pathological significance. Figure 2 displays the amplification curves for all cases and regions interrogated in this study. Individual curves display the mean r.f.u values across triplicate repeats for each case. Several key aggregation kinetics could be resolved and subsequently quantified from these amplification curves. MSA cases had a shorter lag phase, greater AUC, and greater maximum fluorescence than PD cases across all regions (Fig. 2, Figs. S3, S4, S5, S6). PD cases had a longer mean lag time across regions than MSA cases (Table 3, Figs. S4 and S5). Data are the triplicate mean for each case. Representative individual repeats are displayed in Figure S7.

**Table 3:** Summary of statistics for each PD and MSA region

Parameter	Condition	All regions*	Medulla	Substantia nigra	Hippocampus	MTG	Cerebellum
AUC	**PD**	929,582	854,696	1,117,823	1,120,594	644,040	**–**
	**MSA**	2,704,307	2,824,029	2,718,121	2,397,355	**–**	2,877,725
Lag time (h)	**PD**	16.0 ± 5.6	11.9 ± 2.1	16.6 ± 5.1	19.4 ± 6.3	22.3 ± 6.6	**–**
	**MSA**	5.07 ± 1.8	4.4 ± 1.2	4.2 ± 1.8	6.7 ± 1.1	**–**	3.4 ± 1.2
PAR (h^−1^)	**PD**	0.06 ± 0.02	0.086 ± 0.02	0.065 ± 0.02	0.056 ± 0.02	0.049 ± 0.02	**–**
	**MSA**	0.25 ± 0.09	0.24 ± 0.06	0.27 ± 0.08	0.15 ± 0.03	**–**	0.32 ± 0.09
MRF	**PD**	26 ± 11	18 ± 5	29 ± 7	31 ± 15	25 ± 11	**–**
	**MSA**	47 ± 12	51 ± 15	47 ± 13	48 ± 9	**–**	43 ± 12
Gradient	**PD**	3494 ± 2475	3639 ± 2346	4471 ± 2414	3756 ± 3107	2209 ± 1626	**–**
	**MSA**	16,152 ± 9649	14,750 ± 5975	13,594 ± 8326	10,917 ± 5110	**–**	25,345 ± 11,769

Data presented as mean ± SD. PAR, protein aggregation rate; MRF, maximum relative fluorescence; MTG, middle temporal gyrus. * Only regions common to both PD and MSA (medulla, substantia nigra, and hippocampus) were included for quantification

**Fig. 3:**
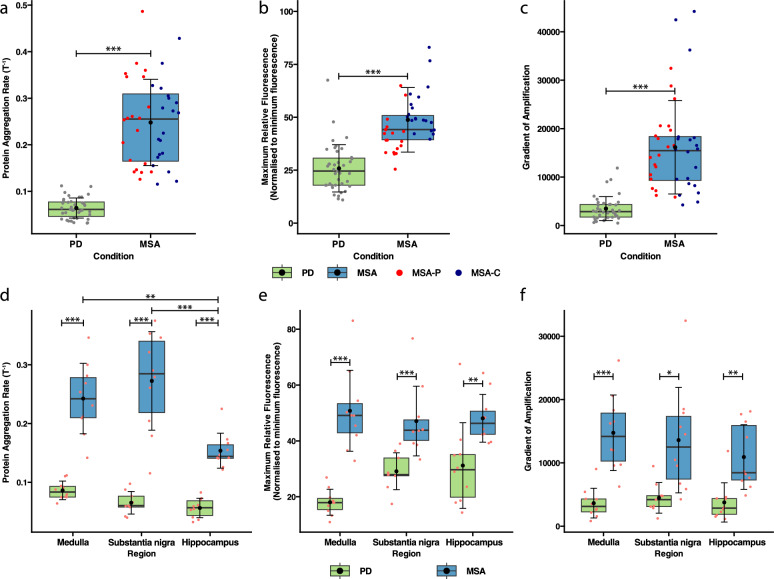
Comparison of key proteoform-specific aggregation kinetics between PD and MSA. **a** Protein aggregation rate (PAR) across all regions. PAR was defined as the inverse of the lag phase (T^−1^), which is the time required to reach the amplification threshold (set to 10^3.5^ r.f.u). Red data points denote individual MSA-P cases, and navy data points denote individual MSA-C cases. **b** Maximum relative fluorescence across all regions. Maximum relative fluorescence was calculated as the maximum/minimum fluorescence ratio. **c** Gradient of amplification across all regions. The gradient of amplification was determined by calculating the gradient of the tangent at the inflection point of each amplification curve. **d**–**f** Comparison of PAR (**d)**, maximum relative fluorescence (**e)**, and gradient of amplification (**f)** within specific brain regions (medulla, substantia nigra, and hippocampus). Filled circles (black) denote the mean kinetic values, the crossbars denote the median kinetic values, and the box plot extremities denote the interquartile range. ***P < 0.001, **P < 0.01, *P < 0.05; Welch’s *t*-test **a–f**, one-way ANOVA with Tukey’s multiple comparison adjustment used to compare between regions **d–f**

The mean PAR, maximum relative fluorescence, and gradient of amplification were significantly higher in MSA cases compared to PD cases across all regions (*P* < 0.001; Fig. 3a–c). These key kinetic differences were also conserved within individual regions. The mean PAR was significantly higher in MSA cases compared to PD cases in the medulla, the substantia nigra, and the hippocampus (*P* < 0.001; Fig. 3d). Among the regions, the middle temporal gyrus had the lowest PAR in PD, and the hippocampus had the lowest PAR in MSA. The medulla was the region with the highest PAR in PD, and the cerebellum had the highest PAR in MSA (Fig. 3d, Table 3).

The maximum relative fluorescence was higher in MSA cases compared to PD cases in the medulla (*P* < 0.001), the substantia nigra (*P* < 0.001), and  the hippocampus (*P* < 0.01; Fig. 3e, Table 3). In MSA, the medulla had the highest maximum relative fluorescence, whereas the cerebellum had the lowest. In contrast, in PD, the hippocampus had the highest maximum relative fluorescence, whereas the medulla had the lowest (Fig. 3e, Table 3).

The mean gradient was higher in MSA cases compared to PD cases in the medulla (*P* < 0.001), the substantia nigra (*P* < 0.05), and hippocampus (*P* < 0.01; Fig. 3f, Table 3). Among the regions, the middle temporal gyrus and the hippocampus had the lowest mean gradient in PD and MSA, respectively, while the substantia nigra and the cerebellum had the highest mean gradient in PD and MSA, respectively (Fig. 3f, Table 3). It is important to note that we did not observe any correlation between the α-Syn aggregation kinetics assessed in this study and any case-specific variables detailed in Table S1.

No significant difference was observed in PAR, maximum relative fluorescence, or gradient between the MSA-P and the MSA-C subtypes within individual regions. However, the MSA-C cases had a higher maximum relative fluorescence than MSA-P across all regions when all cases were grouped for analysis (*P* < 0.05; Table S2, Fig. S8).

### Conformational variability of amplified α-Syn between PD and MSA

**Fig. 4:**
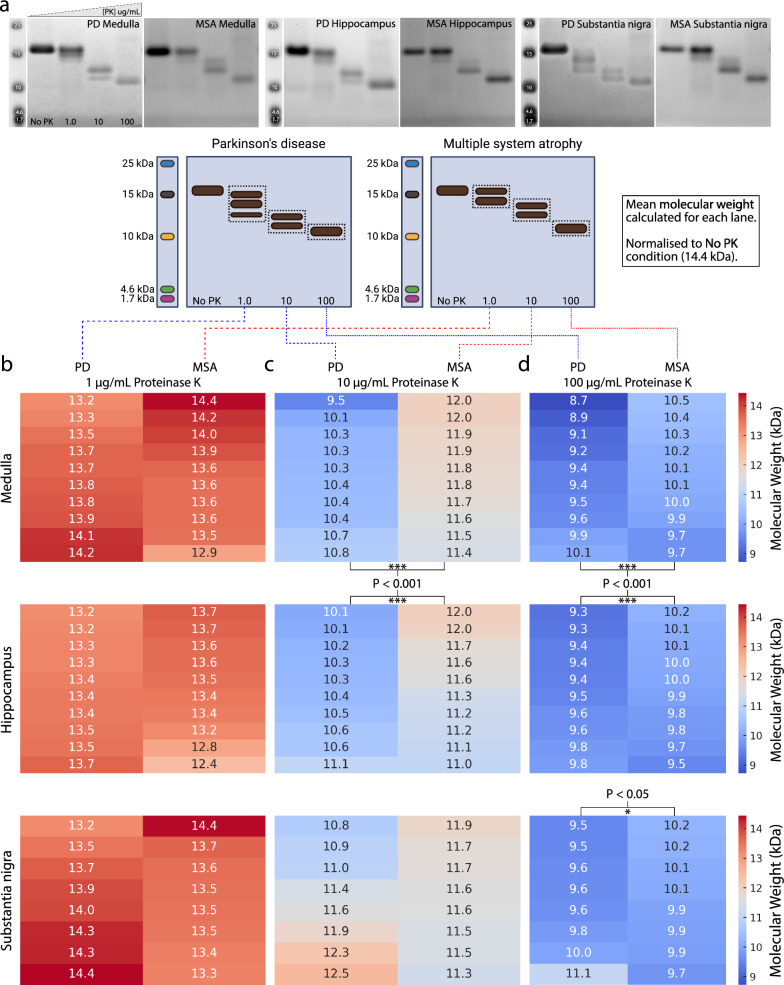
Conformational variability between PD and MSA patient-amplified α-synuclein following proteolytic digestion with proteinase K (PK). **a** Comparison of region-specific α-Syn conformational profiles between PD and MSA. Blots shown are representative and correspond to different cases. The complete set of blots is shown in Figs. S9 and S10. **b–d** Heat maps showing the calculated molecular weights of PK-digested α-Syn proteoforms from the medulla (top row), hippocampus (middle row), and substantia nigra (bottom row) of the PD and MSA patients. Each heat map represents the calculated molecular weight of PK-digested α-Syn for individual PD cases (first column) and MSA cases (second column). The heat maps are organised based on the concentration of PK used for digestion: **b** 1 µg/mL PK, **c** 10 µg/mL PK, **d** 100 µg/mL PK. ***P < 0.001, *P < 0.05; unpaired *t*-test

To investigate the conformational profile of α-Syn in different brain regions in PD and MSA, amplified samples were subjected to proteolytic digestion with PK enzyme. The digestion of α-Syn aggregates by PK varies depending on the conformation-induced exposure of an aggregate to the enzyme. Those regions of the α-Syn protein conformationally exposed at the periphery of the aggregate will be preferentially digested, eventually exposing more central regions of the aggregate for digestion. As an indication of conformational differences, four PK digestion profiles (0, 1, 10, and 100 μg/mL of PK) of PD- and MSA-seeded amplification products across regions are displayed in Fig. S9 (PD) and Fig. S10 (MSA). A representative set of blots comparing the PK digestion profiles of PD- and MSA-seeded amplification products in the medulla, hippocampus, and substantia nigra is shown in Fig. 4a.

Within individual regions, the conformational profile of α-Syn was relatively consistent in both PD and MSA (Figs. S9 and S10). Across regions, greater conformational heterogeneity was observed in PD than in MSA, with the PD middle temporal gyrus and substantia nigra yielding conformational profiles that were distinct from the medulla and hippocampus. Specifically, in the PD medulla and hippocampus, two distinct bands prevailed following digestion with 1 μg/mL and 10 μg/mL PK, whereas three bands were commonly observed in the middle temporal gyrus following digestion with 1 μg/mL PK, whilst three bands were commonly observed in the substantia nigra following digestion with 10 μg/mL PK (Fig. 4a, Fig. S9). In contrast to PD, conformational profiles were relatively conserved across different MSA regions (Fig. S10). Generally, MSA cases yielded two distinct bands following digestion with 1 μg/mL and 10 μg/mL PK (Fig. 4a, Fig. S10); there were, however, infrequent exceptions in which a single band, or sometimes three bands, were detected in the 1 μg/mL condition (Fig. S10). Notably, clear differences were observed for the 10 μg/mL condition between PD and MSA; the highest molecular-weight band of PD cases was typically observed at the same molecular weight as the lowest molecular-weight band of MSA cases. No apparent differences were observed between the MSA-P and MSA-C subtypes within regions (Fig. S10), which was somewhat surprising given the differences in their regional SAA assay outcomes (suggestive of some seeding conformation differences) and the distinct clinical MSA phenotypes associated with their different regional patterns of pathology.

To quantify interregional differences between the conformational profiles of PD and MSA patient-derived α-Syn, molecular weight analysis of digested α-Syn was performed. Briefly, the molecular weight of all bands in each PK concentration condition was quantified and normalised to the molecular weight of the band in the 0 µg/mL PK condition, which was set to 14.4 kDa, the calculated molecular weight of monomeric α-Syn. The molecular weights were averaged if multiple bands were present in a lane (this was the case for the 1 and 10 µg/mL PK lanes). This approach ensured two things: (1) that all cases were internally normalised, which mitigated any interexperimental variability, and (2) that the total number of bands in each condition was considered in the final molecular weight calculation for each lane. A heat map comparing differences in the mean molecular weight between PD and MSA regions for each concentration point is shown in Fig. 4.

The mean molecular weight of α-Syn from the PD medulla following digestion with 10 μg/mL PK was 10.31 ± 0.35 kDa, which was significantly less than the mean molecular weight of α-Syn from the MSA medulla (11.75 ± 0.21 kDa; *P* < 0.001; Fig. 4). Similarly, the mean molecular weight of α-Syn from the PD medulla following digestion with 100 μg/mL PK was 9.40 ± 0.42 kDa, which was significantly less than that of α-Syn from the MSA medulla (10.10 ± 0.27 kDa, *P* < 0.001; Fig. 4). The same was true of the hippocampus, with the mean molecular weight of hippocampal α-Syn in PD following digestion with 10 μg/mL PK being 10.41 ± 0.29 kDa, which was significantly less than the mean molecular weight of α-Syn from the MSA hippocampus (11.48 ± 0.36 kDa, *P* < 0.001; Fig. 4). Similarly, the mean molecular weight of PD hippocampal α-Syn following digestion with 100 μg/mL PK was 9.51 ± 0.20 kDa, which was significantly less than that of α-Syn from the MSA hippocampus (9.91 ± 0.21 kDa, *P* < 0.001; Fig. 4). Finally, the mean molecular weight of α-Syn from the PD substantia nigra following 100 μg/mL PK was 9.84 ± 0.52, which was significantly less than the mean molecular weight of α-Syn from the MSA substantia nigra (10.01 ± 0.16 kDa, *P* < 0.05; Fig. 4). Table S3 displays the proteolytic digestion values for each individual PD and MSA case across interrogated brain regions.

### α-Syn seed amplification from patient-derived CSF

Amplification and subsequent PK digestion of patient-derived α-Syn from brain samples provides crucial insights into the differential seeding propensities and conformational structures of α-Syn in different post-mortem brain regions. The accessibility of patient-derived biofluids during a patient’s lifetime confers several advantages, including the potential to be used in early diagnostics and the ability to monitor seeding and conformational properties throughout the duration of the disease. To this end, seed amplification assays have become increasingly utilised to interrogate the presence of aggregated α-Syn in various patient-derived biosamples, including CSF (reviewed in [39]), nasal swabs [26, 40, 41], skin biopsies [29, 42–45], and serum [46].

**Fig. 5:**
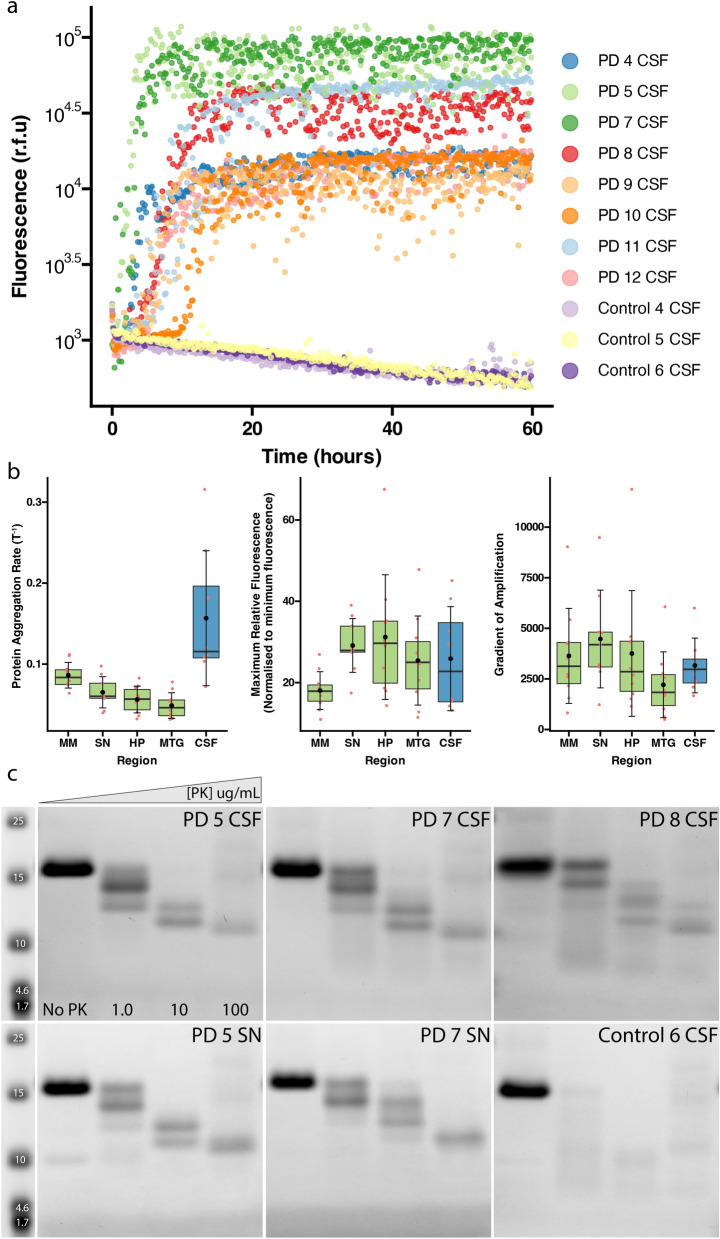
RT-QuIC amplification curves and PK digestion profiles from PD patient-derived CSF. **a** RT-QuIC amplification curves from PD CSF samples (*n* = 8) and neurologically normal CSF samples (*n* = 3). **b** Comparison of PAR, maximum relative fluorescence, and gradient of amplification across PD brain tissue regions and PD CSF samples. Filled circles (black) denote the mean kinetic values, the crossbars denote the median kinetic values, and the box plot extremities denote the interquartile range. **c** PK digestion profiles for PD patient-derived CSF samples. MM, middle medulla; SN, substantia nigra; HP, hippocampus; MTG, middle temporal gyrus; CSF, cerebrospinal fluid; PAR, protein aggregation rate

As a proof-of-concept, PD patient-derived CSF samples were analysed through the same pipeline to detect and structurally interrogate the conformation of α-Syn in CSF. Most of the CSF samples were case-matched with tissue homogenates to permit direct comparison between respective brain regions and CSF. As seen in the amplification curves, PD patient-derived CSF contains sufficient levels of aggregated α-Syn to induce amplification via RT-QuIC using our protocol. In our hands, small amounts of blood contamination did not affect CSF seeding capability (Fig. 5a, PD CSF, *n* = 8). Crucially, none of the neurologically normal CSF samples induced aggregation (Fig. 5a, *n*=3). Figure 5b displays the aggregation kinetics for each PD brain tissue region analysed and compares these values to those obtained when PD CSF samples were amplified. The raw values and the mean ± SD for all patient-matched tissue region and CSF samples are provided in Table S4. CSF samples had a significantly higher PAR than PD brain tissue samples from the medulla (*P* < 0.005), substantia nigra (*P* < 0.001), hippocampus (*P* < 0.0001), and middle temporal gyrus (*P* < 0.0001). No other statistically significant differences were observed between PD brain tissue and CSF samples. Representative PK-digested reaction products from the patient-derived tissue homogenates and CSF (run simultaneously) were comparable, indicating that CSF and brain-derived samples provide the same result using our RT-QuIC protocol (Fig. 5c, PD 5 & 7 CSF compared to SN).

## Discussion

PD and MSA are both neurodegenerative diseases characterised by aberrant accumulation of misfolded α-Syn protein [1–8]. Unlike PD, MSA is an aggressive, fast-progressing, fatal neurodegenerative disease. Their differentiation is, therefore, vital for prognosis, treatment response, and survival [47]. Owing to the similarity among early clinical presentations of α-synucleinopathies, accurate early diagnosis is very challenging, burdened by a misdiagnosis rate of 42% [48] and 82% [49] for PD and MSA, respectively. As such, the potential of SAAs to reliably diagnose α-synucleinopathies early in their pathogenesis cannot be understated, especially in light of recent studies indicating the biological quantitative potential of SAAs beyond a yes/no diagnosis [14, 30, 31].

This study aimed to validate the ability of our RT-QuIC protocol to distinguish between brain tissue homogenates from neuropathologically confirmed PD and MSA cases. This is crucial for assessing the diagnostic accuracy and biological relevance of RT-QuIC, and to minimise the rate of clinical misdiagnosis [48–50]. Additionally, it establishes standardised baseline criteria for disease-specific aggregation kinetics, ensuring that future samples from undiagnosed patients can be accurately compared with validated disease-specific baseline criteria. Importantly, our data demonstrate comparable aggregation kinetics between PD brain tissue homogenates and case-matched CSF samples, highlighting that our RT-QuIC protocol is compatible with detecting α-Syn and quantifying proteoform-specific aggregation kinetics in PD CSF samples. Unfortunately, CSF samples from the MSA cases used in this study were unavailable, precluding comparative investigations of PD versus MSA CSF.

Our RT-QuIC protocol generates visually distinct RT-QuIC response curves separating PD from MSA. Calculating the proteoform-specific aggregation kinetics, including PAR, maximum relative fluorescence, and the gradient of amplification, permits the quantification of disease-specific differences and indicates that these two diseases can be discriminated using RT-QuIC. MSA cases yielded significantly higher values than PD across all three kinetic parameters in brain tissue. Statistical significance was maintained when the data were analysed regionally and when all regions were grouped. In most published studies, the successful differentiation of PD from MSA has not been possible based on these key aggregation kinetics [26, 27, 51]. The higher PAR (shorter lag time) of MSA brain tissue relative to the PD tissue reported here aligns with previous findings [24, 26].

**Fig. 6:**
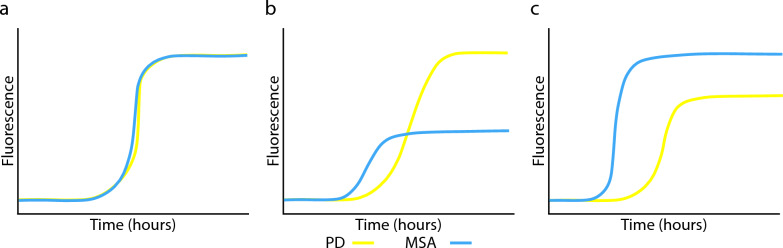
Summary of PD and MSA aggregation kinetics in previous studies compared to the current study. **a** Unable to discriminate between PD and MSA. **b** Previous studies demonstrating that MSA α-Syn yields a greater protein aggregation rate (shorter lag time) and lower maximum relative fluorescence than PD. **c** Present study demonstrating that MSA α-Syn yields a greater protein aggregation rate (shorter lag time) and higher maximum relative fluorescence than PD, reflecting the enhanced biological seeding potency of MSA α-Syn proteoforms

Our demonstration of higher maximum relative fluorescence in MSA relative to PD contradicts some previous studies [24, 52, 53], which report a higher maximum relative fluorescence for PD (Fig. 6b). This discrepancy could be due to the His-tag-modified α-Syn protein used as monomeric substrate and the unique buffer conditions in their SAA protocol [52]. In addition to the seeding protofilament, the α-Syn substrate plays a significant role in the aggregation kinetics and allows selective aggregation of MSA or PD protofilaments [54]. Alterations in the α-Syn monomer significantly impact aggregation kinetics, with specific deletions (such as amino acids 1–19) slowing aggregation and others (like 1–29 or 101–140) increasing it [55]. Removing amino acids 1–54 or 91–140 halts aggregation entirely [55–57]. Disease-related mutations, such as A53T and A30P, lead to faster aggregation and enhance seeding capacity in truncated α-Syn forms [58]. The impact of a C-terminal His-tag on aggregation kinetics is unclear but may affect protein conformation or stability. Further research is needed to determine its effect on the RT-QuIC assay sensitivity for detecting synucleinopathies.

Furthermore, a systematic evaluation of 168 different reaction buffers revealed that the buffer composition can be adjusted to favour the growth of PD or MSA α-Syn strains preferentially [23]. Sarkosyl, a detergent used in some SAA protocols, is worthy of mention. PD α-Syn filaments are less resistant to sarkosyl than MSA [59]. Combined with sonication or shaking, this causes an enhanced release from PD filaments while maintaining seeding activity throughout the seed amplification assay [59]. Utilising sarkosyl in SAAs would theoretically lead to accelerated aggregation kinetics for PD. While including sarkosyl in the SAA reaction buffers allows separation between PD and MSA, the resulting kinetic readout demonstrating a higher maximum fluorescence for PD than MSA (Fig. 6b) does not reflect the enhanced biological seeding potency of MSA α-Syn proteoforms, with MSA being a more aggressive and faster-progressing disease with a much shorter disease duration than PD [60, 61]. In contrast, SDS used in the present study is a more aggressive detergent than sarkosyl and more effectively denatures and solubilises α-Syn [37]. MSA has substantially higher region-dependant amounts of membrane-associated α-Syn in SDS-soluble fractions than PD (+ 356% to 6830% for MSA vs − 60% to + 618% for PD) [62] and therefore increases the available pool of seed-competent α-Syn from MSA tissue relative to less aggressive homogenisation protocols [23].

While the higher PAR (shorter lag time) of MSA patient-derived α-Syn might also reflect the higher pathology load of MSA cases, our data revealed that MSA seeding activity is insensitive to dilutions of the initial seed concentration, even at minute concentrations (3×10^–5^, Fig. S3a). Even at these very dilute tissue concentrations, MSA cases yielded significantly higher PARs than PD at the lowest dilutions (0.02% *w*/*v* for MSA compared to an end-point dilution of 0.125% for PD, Fig. S3b), suggesting that while pathology load plays a role, MSA patient-derived α-Syn is also a more potent aggregation seed than PD patient-derived α-Syn. The PD-specific sensitivity of its α-Syn seeds to dilution suggests that PD α-Syn has decreased seeding efficiency compared to MSA α-Syn, which is consistent with the decreased ability of PD α-Syn to seed pathological α-Syn conformations in animal and cell models, compared to MSA α-Syn [63]. Based on the available biological data, the expected MSA SAA profile would be characterised by a higher PAR (shorter lag time) and higher maximum fluorescence compared to PD. As a final note on pathology load, we have previously shown that the MSA cases examined in this study exhibit a significantly higher α-Syn pathology load compared to the PD cases across all analysed regions, with the exception of the hippocampus, which had a significantly lower pathology load in MSA than in PD [38]. Although the MSA hippocampus has significantly lower α-Syn pathology load (0.20%) than the PD hippocampus (0.65%) [38], MSA cases still yielded significantly higher aggregation kinetics across all three key parameters than PD cases (Fig. 3d–f). Interestingly, the PAR for MSA hippocampal α-Syn was still significantly lower than that for the MSA medulla and substantia nigra (Fig. 3d), which suggests that α-Syn pathology load does play a role in driving PAR. However, its influence is likely secondary to the inherent biological properties of disease-specific strains.

Collectively, the differential proteoform-specific aggregation kinetics reported in this study suggest four key distinctions between α-Syn derived from PD and MSA human brain tissues while retaining the characteristics observed in the disease (summarised in Fig. 6). (1) The higher PAR (shorter lag time) of MSA patient-derived α-Syn suggests that it is a more potent aggregation seed than PD patient-derived α-Syn. (2) The higher PAR of MSA patient-derived α-Syn is also influenced by the higher α-Syn pathology load in MSA. (3) The higher maximum relative fluorescence of MSA patient-derived α-Syn could reflect a higher binding affinity of MSA α-Syn for Thioflavin-T, indicating a conformational difference between PD and MSA patient-derived α-Syn [11, 39, 64, 65]. (4) The higher gradient of amplification of MSA patient-derived α-Syn suggests that it has a more potent ability to seed fibrillar α-Syn growth once the initial aggregation threshold has been reached, thereby achieving a more rapid exponential growth phase [39].

While various RT-QuIC protocols can differentiate between PD and MSA (Fig. 6), we stress that careful selection of buffer and substrate composition is crucial for generating biologically relevant SAA outputs. A key unknown is whether the seeds amplified by SAA maintain the same configuration as those found in the brain, as determined with cryogenic electron microscopy. Notably, the purification process used to generate specimens for cryogenic electron microscopic analysis is designed to extract high molecular weight fibrils, potentially losing smaller prefibrillar and oligomeric material that can be amplified by SAAs [66]. This distinction is crucial, given that α-Syn inclusions in MSA consist of two types of filaments, each containing two distinct protofilaments [10]. The structures of α-Syn filaments extracted from the brains of individuals with MSA differ from those formed in vitro [11]. These differences have significant implications for understanding the mechanisms of aggregate propagation and the resulting neurodegeneration, emphasising the need for protocols that preserve the biological relevance of the disease [10].

Theranostic SAAs present an exciting opportunity to bridge the gap between diagnostics and therapeutics for neurodegenerative diseases. For instance, SAAs can be used to assess the efficacy of therapeutic compounds that aim to return pathological α-Syn to a more stable, less aggregation-prone protofilament state through real-time analysis of PAR and maximum relative fluorescence. In this theranostic capacity, patient-derived biosamples could be (1) analysed to obtain an initial diagnosis and (2) simultaneously amplified with a range of potential therapeutic compounds designed to reduce the seeding potential of patient-derived α-Syn strains. This theranostic approach would also permit patient-matched therapeutic screening in vitro and could provide information on which potential therapeutic compounds might be the most efficacious for an individual patient. The utility of SAAs as a theranostic methodology could be invaluable in the initial screening and development of disease-modifying compounds and in assessing their patient-specific efficacy. Importantly, even if these therapeutic compounds cannot completely inhibit the seeding capacity of patient-derived α-Syn strains, their potential to slow the seeding capacity of pathological α-Syn would still be of great therapeutic benefit. These inhibitory compounds could then form the basis for developing systemic drugs to slow the progression of both PD and MSA.

Our proteolytic digestion protocol provides additional biological relevance to the RT-QuIC protocol. The main advantage of proteolysis with PK over other proteases used to isolate amyloid cores is its low amino acid specificity, which allows near-complete hydrolysis of non-core regions [67]. This is important as the core regions of the protofilaments govern α-Syn aggregation [54]. Our proteolytic digestion data demonstrate significant differences in the digestion profile of PD and MSA patient-derived α-Syn, with MSA α-Syn core protofilaments generally exhibiting a greater resistance to PK digestion in all investigated brain regions (medulla, hippocampus, and substantia nigra).

Interestingly, while we observed region-specific digestion profiles in PD, we did not observe any region-specific digestion profiles in MSA. These data largely align with previous findings by Strohäker et al*.* (2019), who showed that α-Syn fibrils propagated from PD patients exhibit greater structural heterogeneity than fibrils propagated from MSA [11]. While the distinct digestion profiles of PD and MSA could indicate disease-specific strains, more extensive structural interrogations with electron microscopy, nuclear magnetic resonance spectroscopy, and electron paramagnetic resonance, which were beyond the scope of the present study, are required.

Both MSA-P and MSA-C patient samples exhibited clear seeding activity with our protocol. It is important to highlight that previous studies, by and large, do not stratify the two disease subtypes, and those that do are highly incongruent [68, 69]. To this end, Bargar et al*.* (2021) previously reported that olfactory mucosa samples from MSA-P cases seeded α-Syn aggregation with RT-QuIC, but MSA-C samples did not [69]. In contrast, Poggiolini et al*.* (2021) showed that 100% of CSF samples from MSA-C cases induced α-Syn aggregation with RT-QuIC, while only 57% of MSA-P cases did so [51]. The lack of any difference in the proteolytic digestion profiles between MSA-P and MSA-C cases in the present study aligns with previous findings by Okuzumi et al*.* (2023), who showed no morphological differences between these two subtypes [46]. While our protocol suggests that RT-QuIC can indeed be used to discriminate MSA subtypes, with MSA-C cases yielding a significantly higher maximum relative fluorescence than MSA-P when all regions were combined, further investigations with more subtype-specific cases are needed to confirm this.

## Conclusions

Our findings show that PD and MSA (both MSA-P and MSA-C) can be identified using our RT-QuIC protocol and subsequent quantification of proteoform-specific aggregation kinetics and proteolytic digestion assay. MSA α-Syn propagates more rapidly and exhibits higher maximum relative fluorescence and gradient of amplification, distinguishing it from PD α-Syn in analysed brain regions. This distinction is pivotal, as it may reflect intrinsic pathological differences between the diseases and the differential RT-QuIC aggregation kinetics could reflect the biological disease features. We propose that with additional validation, the SAA protocol could evolve from a purely diagnostic to a theranostic biomarker that can be used as a measure to help develop new therapeutics.

## Supplementary Information

**Additional file 1.** **Table S1.** Case information for human brain tissue used in this study. **Table S2.** Summary of statistics for each MSA-P vs MSA-C comparison for each region. **Table S3. **Concentration-dependent proteolytic digestion values for each region across individual PD and MSA cases. **Table S4. **Comparison of RT-QuIC aggregation kinetics between PD brain tissue regions and PD CSF samples. **Figure S1.** Representative RT-QuIC amplification curves from neurologically normal control brain tissue. **Figure S2.** Flow chart detailing the key aspects involved in optimising the RT-QuIC and PK digestion workflow. **Figure** **S3**. MSA patient-derived α-Syn seeding activity maintains robust insensitivity to dilutions in the initial seed concentration, whereas PD patient-derived α-Syn seeding capacity was considerably reduced at lower initial seed concentrations. **Figure S4.** Regional comparison of lag phase and AUC in PD and MSA. **Figure S5.** Case-specific comparison of lag phase and AUC in PD and MSA. **Figure S6.** Comparison of AUC between PD and MSA. **Figure S7.** Representative RT-QuIC amplification curves for individual triplicate repeats across common PD and MSA regions. **Figure S8. **Comparison of maximum relative fluorescence between MSA-P and MSA-C. **Figure S9.** Conformational profiles of PD patient-derived α-Syn following PK digestion. **Figure S10.** Conformational profiles of MSA patient-derived α-Syn following PK digestion.

## Data Availability

All data are available in the main text, or in the Supplementary Tables and Supplementary Figures. The raw data that support the findings of this study are available from the corresponding author upon reasonable request.

## Abbreviations

α-Syn: Alpha-synuclein
ANOVA: Analysis of variance
AUC: Area under the curve
CSF: Cerebrospinal fluid
MRF: Maximum relative fluorescence
MSA: Multiple system atrophy
MSA-C: MSA-cerebellar phenotype
MSA-P: MSA-parkinsonian phenotype
MTG: Middle temporal gyrus
PAR: Protein aggregation rate
PBS: Phosphate buffered saline
PD: Parkinson’s disease
PK: Proteinase K
r.f.u: Relative fluorescent units
RT-QuIC: Real-time quaking-induced conversion
SAA: Seed amplification assay
SD: Standard deviation
