# Supplementary material for: Refining α-synuclein seed amplification assays to distinguish Parkinson’s disease from multiple system atrophy
Source: Transl Neurodegener. 2025 Feb 7;14:7. doi: 10.1186/s40035-025-00469-6 (PMC11804046; doi:10.1186/s40035-025-00469-6)
Supplement: Supplementary file 1 — Additional file 1. Table S1. Case information for human brain tissue used in this study. Table S2. Summary of statistics for each MSA-P vs MSA-C comparison for each region. Table S3. Concentration-dependent proteolytic digestion values for each region across individual PD and MSA cases. Table S4. Comparison of RT-QuIC aggregation kinetics between PD brain tissue regions and PD CSF samples. Figure S1. Representative RT-QuIC amplification curves from neurologically normal control brain tissue. Figure S2. Flow chart detailing the key aspects involved in optimising the RT-QuIC and PK digestion workflow. Figure S3. MSA patient-derived α-Syn seeding activity maintains robust insensitivity to dilutions in the initial seed concentration, whereas PD patient-derived α-Syn seeding capacity was considerably reduced at lower initial seed concentrations. Figure S4. Regional comparison of lag phase and AUC in PD and MSA. Figure S5. Case-specific comparison of lag phase and AUC in PD and MSA. Figure S6. Comparison of AUC between PD and MSA. Figure S7. Representative RT-QuIC amplification curves for individual triplicate repeats across common PD and MSA regions. Figure S8. Comparison of maximum relative fluorescence between MSA-P and MSA-C. Figure S9. Conformational profiles of PD patient-derived α-Syn following PK digestion. Figure S10. Conformational profiles of MSA patient-derived α-Syn following PK digestion. [file 40035_2025_469_MOESM1_ESM.pdf]

Supplementary Material

Refining α-Synuclein Seed Amplification Assays to Distinguish Parkinson's Disease from Multiple System Atrophy

James A Wiseman[1,2,3], Clinton P Turner[4], Richard L. M. Faull[1,2], Glenda M Halliday[3,5], Birger Victor Dieriks[1,2,3].

1 Department of Anatomy and Medical Imaging, University of Auckland, Auckland, New Zealand

2 Centre for Brain Research, University of Auckland, Auckland, 1023, New Zealand

3 Brain and Mind Centre & Faculty of Medicine and Health School of Medical Sciences, The University of Sydney, Sydney, NSW 2050, Australia

4 LabPlus, Department of Anatomical Pathology, Te Whatu Ora, Auckland, New Zealand

5 Neuroscience Research Australia & Faculty of Medicine School of Medical Sciences, University of New South Wales, Sydney, NSW 2052, Australia

Correspondence to:
Birger Victor Dieriks
Department of Anatomy and Medical Imaging
85 Park Road, Grafton
Auckland 1142
v.dieriks@auckland.ac.nz

Supplementary Tables

Table S1. Case information for human brain tissue used in this study.

**Case**	**Neuropathological Diagnosis**	**Age**	**Sex**	**PMD (h)**	**Cause of Death**	**Duration with Disease (Years)**
MSA 1	MSA-P	61	M	21	Aspiration pneumonia	12
MSA 2	MSA-P	64	M	22	Bronchopneumonia	10
MSA 3	MSA-P	61	M	7	Respiratory arrest	4
MSA 4	MSA-P	84	F	30	Cardiorespiratory failure	22
MSA 5	MSA-P	74	F	18	Sepsis	8
MSA 6	MSA-C	69	M	41	Aspiration pneumonia	14
MSA 7	MSA-C	70	M	45	Pneumonia	7
MSA 8	MSA-C	66	F	15	Cardiorespiratory failure	7
MSA 9	MSA-C	54	M	27	Cardiorespiratory failure	10
MSA 10	MSA-C	74	F	20	Renal failure	7
PD 1	PD	60	F	15.5	Bronchopneumonia/ multisystem organ failure	7
PD 2	PD	78	M	6	Aspiration pneumonia	19
PD 3	PD	80	M	18	Urosepsis	26
PD 4	PD	91	F	5	End-stage PD	22
PD 5	PD	67	M	2.25	End-stage PD and DLB	9
PD 6	PD	73	M	17.5	Aspiration pneumonia	22
PD 7	PD	65	M	17	Bronchopneumonia	12
PD 8	PD	80	M	5.5	Pneumonia	9
PD 9	PD	76	F	6.5	Abdominal carcinoma	23
PD 10	PD	77	M	6.5	End-stage PD	22
PD 11	PD	73	M	14	Congestive heart failure	–
PD 12	PD	84	F	22	End-stage PD	–
Control 1	Neurologically normal	84	M	36	Pulmonary hypertension	N/a
Control 2	Neurologically normal	80	F	29	Cardiac failure	N/a
Control 3	Neurologically normal	89	M	27	Pulmonary embolism	N/a
Control 4	Neurologically normal	63	M	20	Asphyxia	N/a
Control 5	Neurologically normal	93	F	19	Pneumonia	N/a
Control 6	Neurologically normal	77	F	13	Ischaemic heart disease, coronary atherosclerosis	N/a

Cases with both patient-derived brain tissue and CSF are indicated with *. Cases with only CSF are *italicised*. PMD, post-mortem delay.

Table S2. Summary of statistics for each MSA-P vs MSA-C comparison for each region.

**Condition** Parameter	**All regions combined**	**Medulla**	**Substantia nigra**	**Hippocampus**	**Cerebellum**
**MSA-P** Lag time (hr)	5.0 ± 1.8	4.9 ± 1.4	4.0 ± 1.3	7.2 ± 0.4	4.0 ± 1.5
**MSA-C** Lag time (hr)	4.3 ± 1.8	3.9 ± 0.8	4.3 ± 2.4	6.18 ± 1.4	2.82 ± 0.4
**MSA-P** PAR (hr^{-1})	0.23 ± 0.09	0.22 ± 0.06	0.27 ± 0.09	0.14 ± 0.01	0.29 ± 0.12
**MSA-C** PAR (hr^{-1})	0.27 ± 0.09	0.27 ± 0.06	0.27 ± 0.09	0.17 ± 0.04	0.36 ± 0.05
**MSA-P** MRF	43 ± 9.9	48 ± 13.1	40 ± 4.3	47 ± 8.3	35 ± 9.1
**MSA-C** MRF	52 ± 12.4	54 ± 16.6	55 ± 13.9	49 ± 9.6	48 ± 11.2
**MSA-P** Gradient	16420 ± 11639	12,562 ± 4,924	10,823 ± 5,815	11,511 ± 6,026	30,784 ± 14,256
**MSA-C** Gradient	15883 ± 7450	16,938 ± 6,642	16,365 ± 10,147	10,323 ± 4,642	19,907 ± 5,871

Data presented as mean ± SD. PAR, Protein Aggregation Rate; MRF, Maximum Relative Fluorescence.

Table S3. Concentration-dependent proteolytic digestion values for each region across individual PD and MSA cases.

		PD µg/mL			MSA µg/mL		
**Region**	**Case**	**1**	**10**	**100**	**1**	**10**	**100**
**Medulla**	**1**	13.2	10.4	9.5	13.6	11.8	10.1
	**2**	13.3	10.1	9.2	13.9	11.5	10.2
	**3**	13.5	10.4	8.9	12.9	11.9	10.3
	**4**	13.8	10.8	10.1	13.6	12.0	9.9
	**5**	14.1	10.3	9.6	14.4	11.7	10.0
	**6**	14.2	9.5	8.7	14.0	11.4	9.7
	**7**	13.9	10.3	9.4	13.6	11.8	10.1
	**8**	13.7	10.3	9.4	13.5	11.6	10.4
	**9**	13.8	10.4	9.1	13.6	11.9	10.5
	**10**	13.7	10.7	9.9	14.2	12.0	9.7
**Hippocampus**	**1**	13.2	10.1	9.3	13.7	12.0	10.1
	**2**	13.4	10.1	9.4	13.5	11.2	10.0
	**3**	13.2	10.2	9.3	13.6	11.0	10.2
	**4**	13.3	10.3	9.4	13.2	11.7	9.7
	**5**	13.5	10.3	9.4	13.4	11.6	9.8
	**6**	13.3	10.4	9.5	13.4	11.2	9.8
	**7**	13.5	10.5	9.6	12.8	12.0	9.9
	**8**	13.4	10.6	9.6	13.6	11.3	10.1
	**9**	13.4	10.6	9.8	13.7	11.1	10.0
	**10**	13.7	11.1	9.8	12.4	11.6	9.5
**Substantia nigra**	**1**	13.7	11.6	9.5	13.7	11.7	9.9
	**2**	13.2	10.8	9.5	13.5	11.7	10.1
	**5**	14.3	11.0	9.6	13.4	11.6	9.9
	**6**	14.3	10.9	9.6	13.6	11.3	10.2
	**7**	13.9	11.4	10.0	13.5	11.5	9.9
	**8**	13.5	11.9	9.6	13.5	11.6	10.1
	**9**	14.4	12.5	9.8	14.4	11.9	10.2
	**10**	14.0	12.3	11.1	13.3	11.5	9.7

Table S4. Comparison of RT-QuIC aggregation kinetics between PD brain tissue regions and PD CSF samples.

Case	Region	Rate	MRF	Gradient
PD3	HP	0.05	18	1761
PD5	HP	0.06	30	3024
PD7	HP	0.07	40	1515
PD8	HP	0.04	19	4322
PD9	HP	0.03	24	2693
PD10	HP	0.05	29	4593
		(0.05 ± 0.01)	*(26.65 ± 8.47)*	*(2984.62 ± 1274.10)*
PD3	MM	0.07	19	3519
PD5	MM	0.11	18	5424
PD7	MM	0.09	13	2595
PD8	MM	0.08	17	4252
PD9	MM	0.08	15	2155
PD10	MM	0.06	18	4311
		(0.08 ± 0.02)	*(16.56 ± 2.04)*	*(3709.38 ± 1207.42)*
PD5	SN	0.07	39	4193
PD7	SN	0.06	27	1226
PD8	SN	0.04	29	2863
PD9	SN	0.08	17	3195
PD10	SN	0.10	36	6602
		(0.07 ± 0.02)	*(29.80 ± 8.49)*	*(3615.62 ± 1981.55)*
PD3	MTG	0.03	13	624
PD5	MTG	0.03	11	499
PD7	MTG	0.08	27	1714
PD8	MTG	0.05	25	1666
PD9	MTG	0.07	25	6065
PD10	MTG	0.05	18	1021
		(0.05 ± 0.02)	*(19.86 ± 6.83)*	*(1931.56 ± 2087.97)*
PD3	CSF	0.18	14	3913
PD5	CSF	0.32	41	6000
PD7	CSF	0.24	33	2368
PD8	CSF	0.12	29	3333
PD9	CSF	0.11	13	2727
PD10	CSF	0.07	16	3214
		(0.17 ± 0.09)	*(24.39 ± 11.46)*	*(3592 ± 1292.75)*

Mean ± SD for each region is shown in brackets *(italicised)*. Cases for which patient-matched CSF was not available have been omitted.

Supplementary Figures

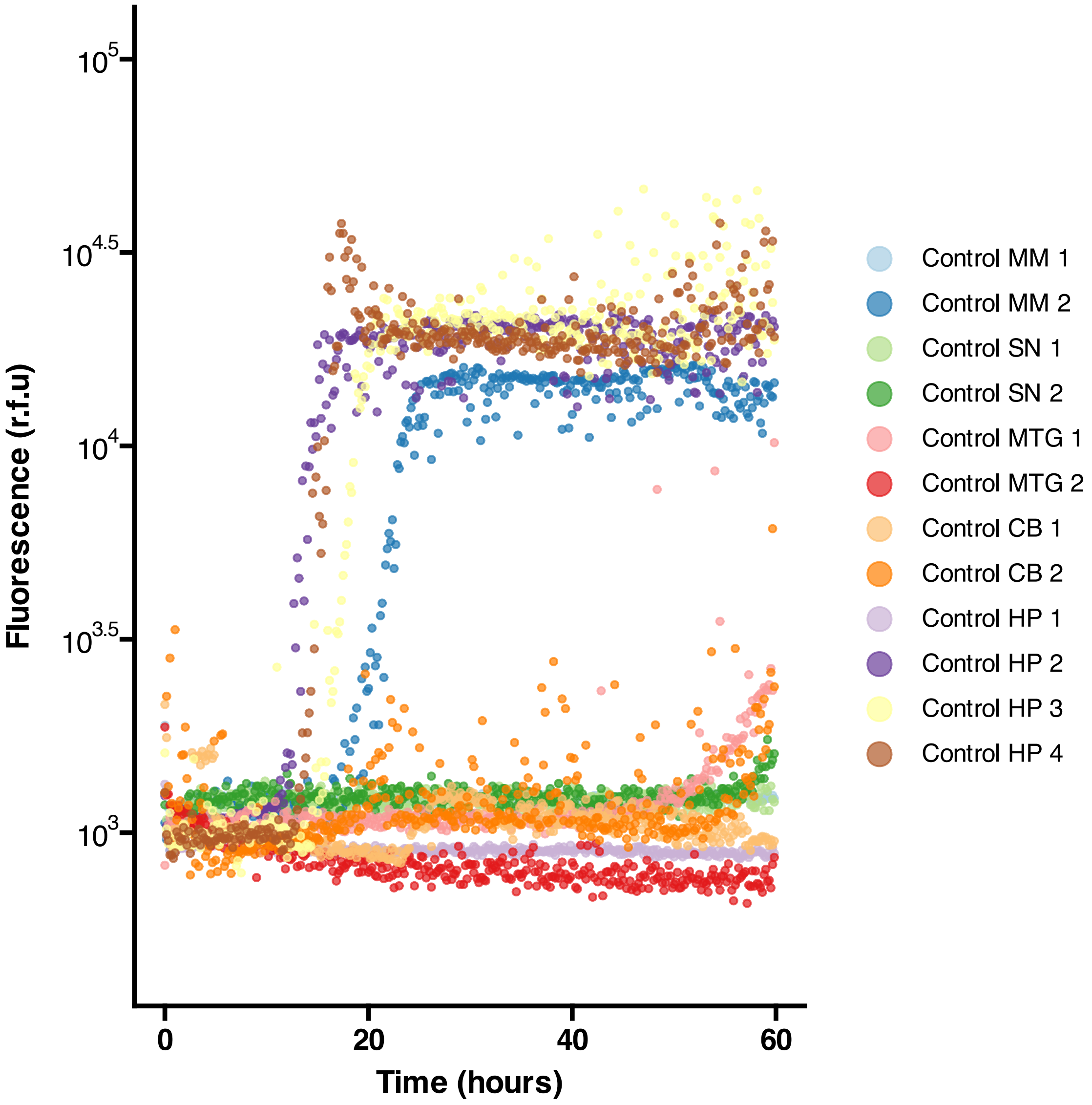

Figure S1. Representative RT-QuIC amplification curves from neurologically normal control brain tissue. All RT-QuIC assays included region-matched neurologically normal control samples and neurologically normal control middle temporal gyrus (MTG) samples. MTG samples were included as there was no significant age-related pathology in this region. Varying levels of sporadic age-related pathology were observed in control hippocampal sections following immunolabelling of control cases. Occasional amplification of α-Syn also occurred when seeded with neurologically normal hippocampus homogenates (older cases > 80 years) and, therefore, pathology-free homogenates from the adjacent cortical regions were included as additional neurologically normal controls.

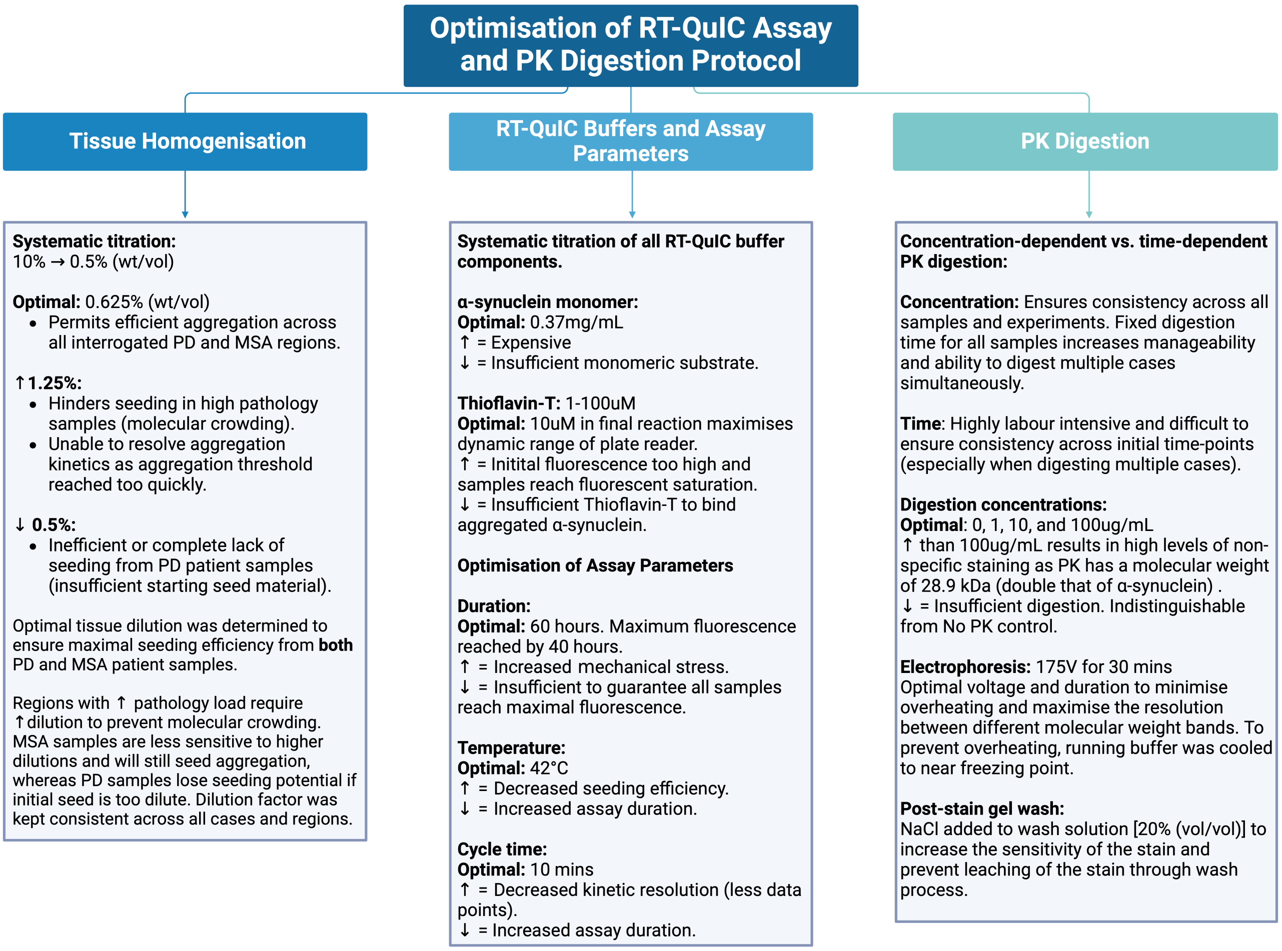

Figure S2. Flow chart detailing the key aspects involved in optimising the RT-QuIC and PK digestion workflow.

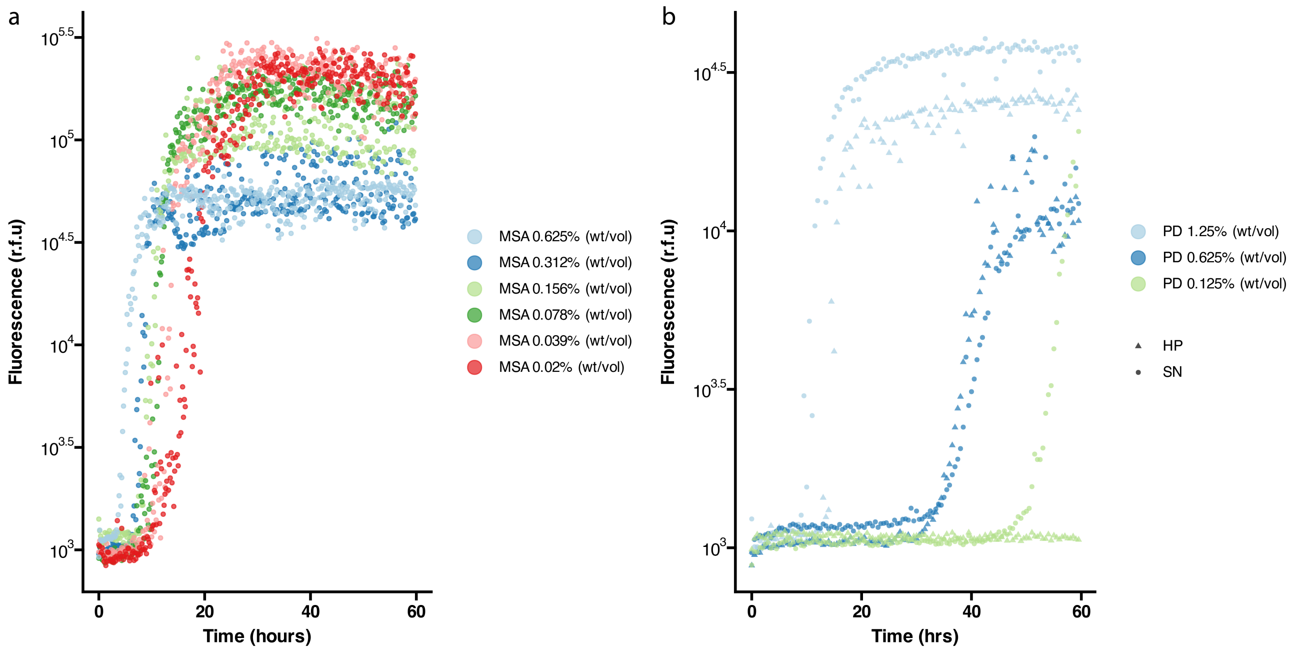

Figure S3. MSA patient-derived α-Syn seeding activity maintains robust insensitivity to dilutions in the initial seed concentration (a), whereas PD patient-derived α-Syn seeding capacity was considerably reduced at lower initial seed concentrations (b, 0.125%).

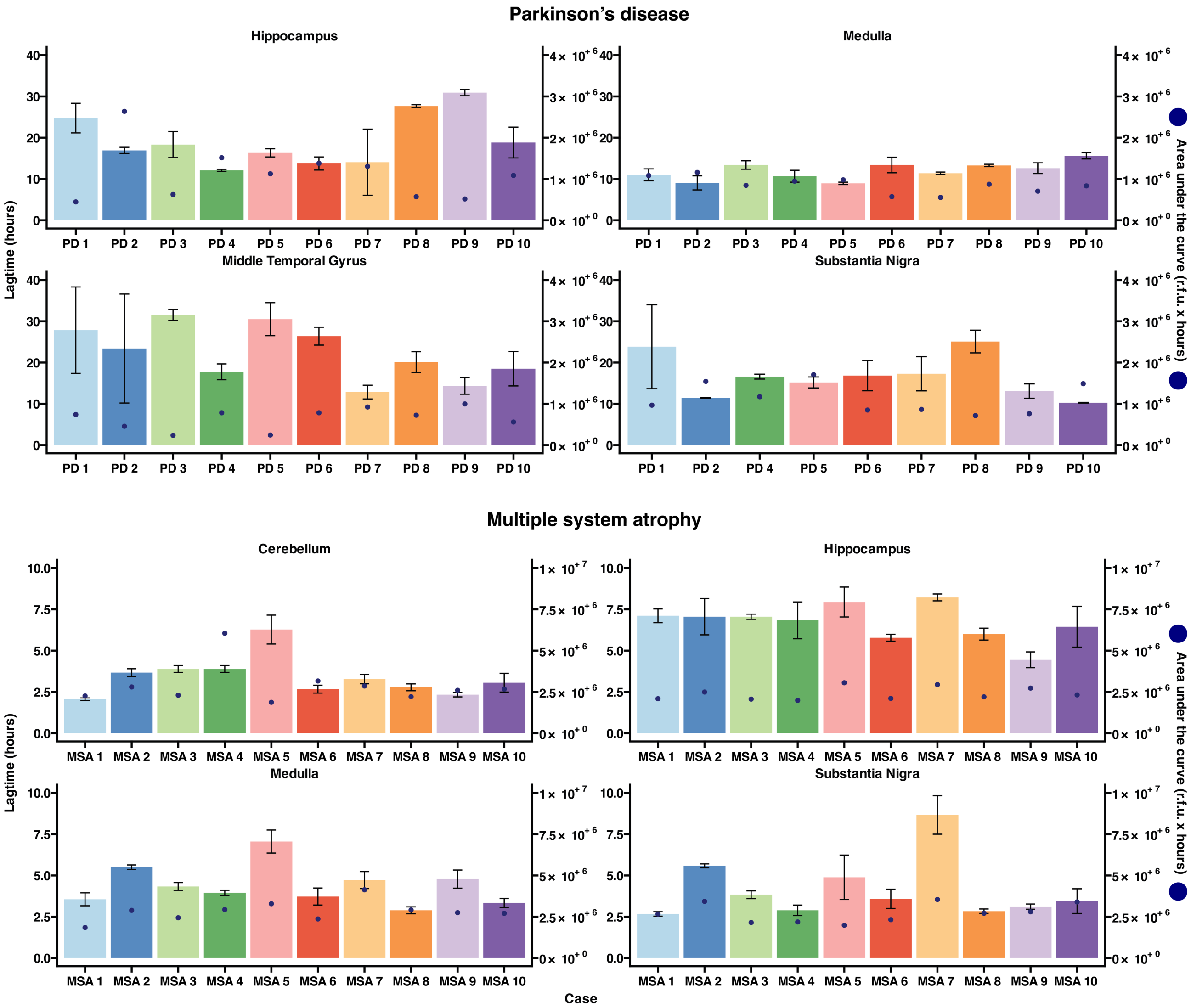

Figure S4. Regional comparison of lag phase and AUC in PD and MSA. Mean lag phase (bars) is plotted on the primary y-axis, and mean AUC (blue dots) is plotted on the secondary y-axis. Lag phase is presented as mean ± SD across all three sample replicates.

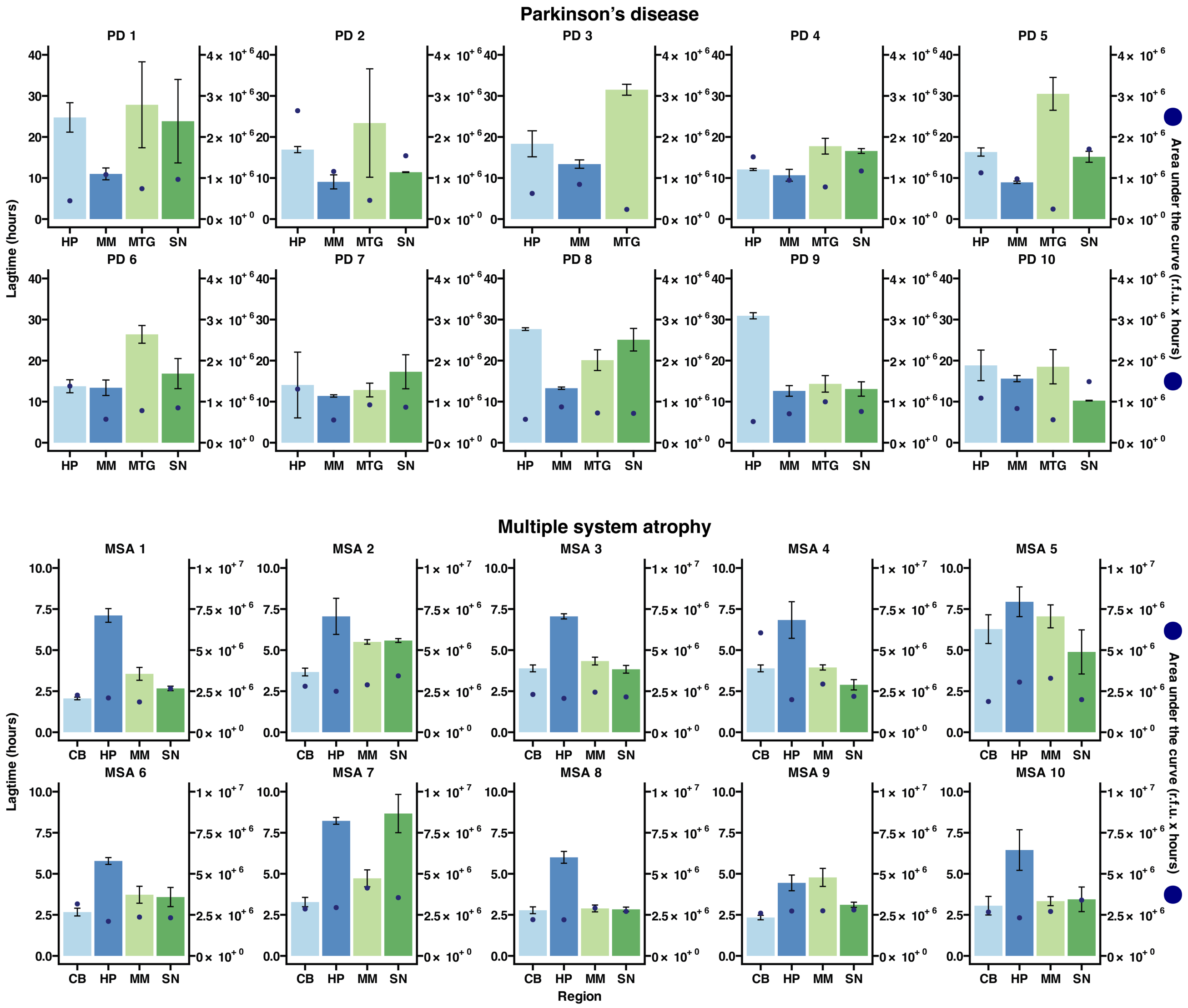

Figure S5. Case-specific comparison of lag phase and AUC in PD and MSA. Mean lag phase (bars) is plotted on the primary y-axis and mean AUC (blue dots) is plotted on the secondary y-axis. Lag phase is presented as mean ± SD across all three sample replicates.

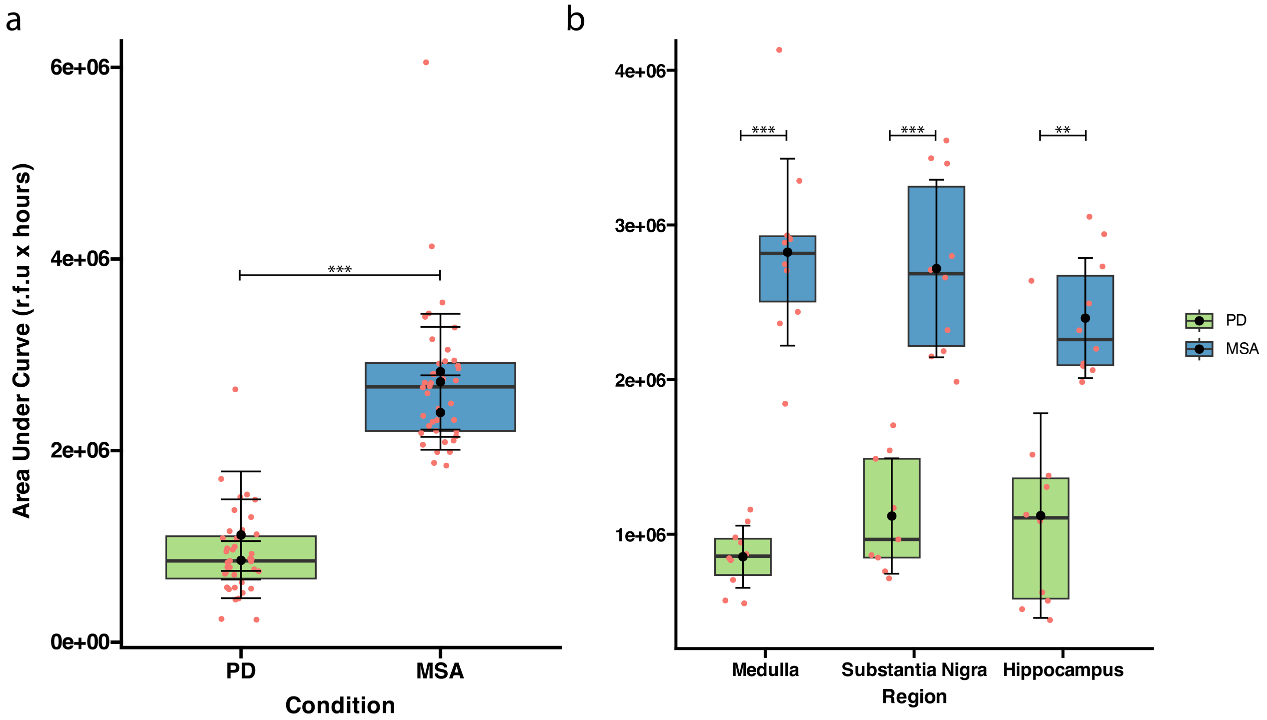

Figure S6. Comparison of AUC between PD and MSA. (a) Mean AUC for PD cases relative to MSA cases. (b) Mean region-specific AUC for PD and MSA cases. Filled circles (black) denote the mean kinetic values, the crossbars denote the median kinetic values, and the box plot extremities denote the interquartile range. *** $p < 0.001$, ** $p < 0.01$, * $p < 0.05$; Mann-Whitney U test.

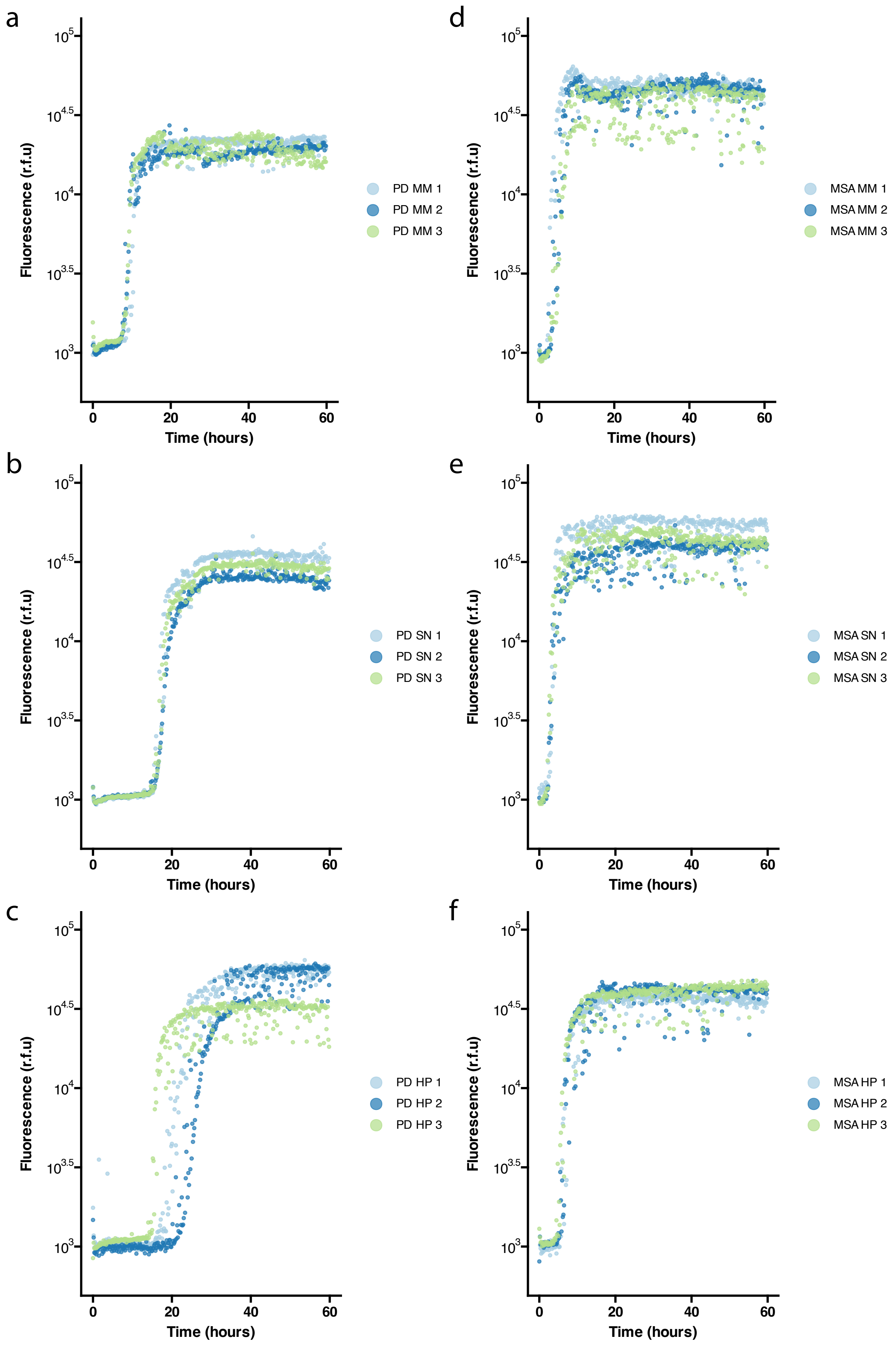

Figure S7. Representative RT-QuIC amplification curves for individual triplicate repeats across common PD and MSA regions.

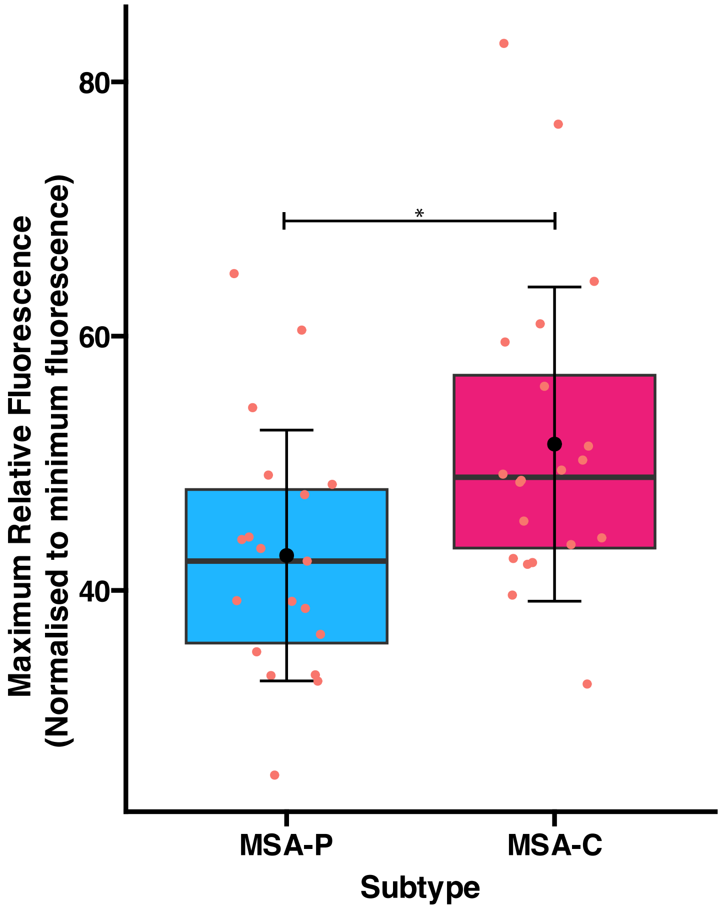

Figure S8. Comparison of maximum relative fluorescence between MSA-P and MSA-C. Filled circles (black) denote the mean kinetic values, the crossbars denote the median kinetic values, and the box plot extremities denote the interquartile range. * $p < 0.05$, Welch's t-test.

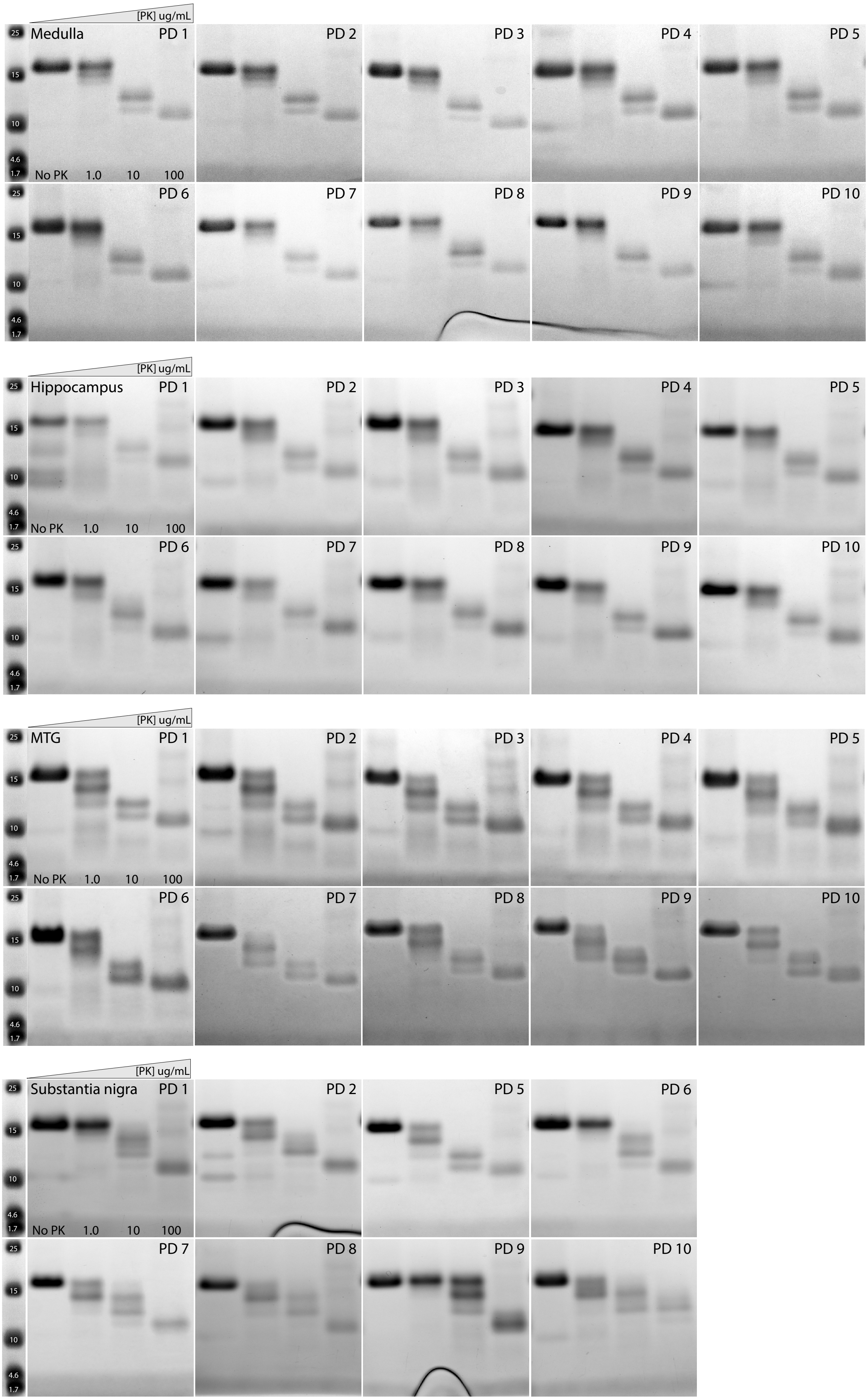

Figure S9. Conformational profiles of PD patient-derived α-Syn following PK digestion.

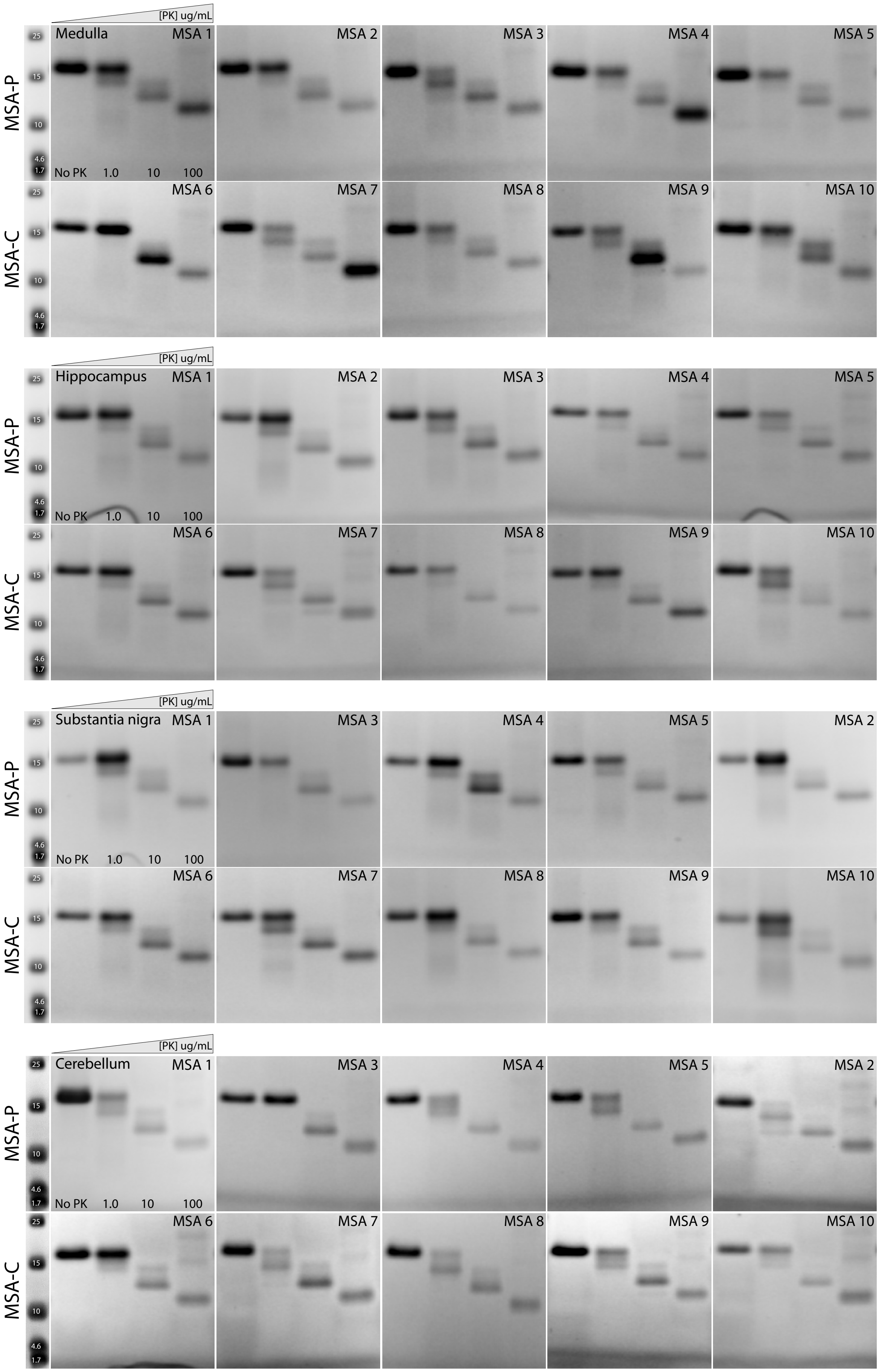

Figure S10. Conformational profiles of MSA patient-derived α-Syn following PK digestion.
